# Associations between parental support and physical activity in children and adolescents with visual impairments: a mediating analysis of exercise self-efficacy

**DOI:** 10.7717/peerj.21871

**Published:** 2026-10-28

**Authors:** Li Dou, Huawei Chen, Enjie Lu

**Affiliations:** 1 Department of Physical Education and Aesthetic Education, Nanjing Forestry University, Nanjing, Jiangsu Province, China; 2 Department of Physical Education, Nanjing University of Aeronautics & Astronautics, Nanjing, Jiangsu Province, China; 3 Department of Physical Education, Nanjing Blind School, Nanjing, Jiangsu Province, China

**Keywords:** Physical activity, Children and adolescents with visual impairments, Parental support, Exercise self-efficacy, Moderate-to-vigorous physical activity

## Abstract

**Purpose:**

Physical activity (PA) is imperative for the physical and mental health of children and adolescents with visual impairments (VI). However, functional impairment often leads to reduced PA, resulting in declines in health and quality of life. Although parental support (PS) has been identified as a key social determinant of PA in typically developing youth, its specific mechanisms, particularly the mediating role of exercise self-efficacy (ESE), remain unclear in children and adolescents with VI. This study aimed to examine the associations between PS and moderate-to-vigorous physical activity (MVPA), as well as the mediating role of ESE, in this population.

**Methods:**

Using a cross-sectional design, 306 children and adolescents with VI aged 7–18 (mean age 13.80 ± 2.94 years) from specialized schools in East China were recruited through convenience sampling from September to December 2024. Accelerometers were used to objectively measure PA levels, and MVPA was extracted for analysis. PS for PA and ESE were assessed using validated scales. The associations between PS and MVPA were analyzed. Structural equation modeling (SEM) and the bias-corrected (BC) percentile bootstrap method were employed to analyze the direct and indirect effects of PS on MVPA, with ESE tested as a mediator.

**Results:**

PS and ESE were both positively correlated with MVPA (*r* = 0.306 and 0.385, respectively; both *P* < 0.05). In the structural equation model, PS had a significant total effect on MVPA (β = 0.379, 95% confidence interval (CI) [0.226–0.523], *P* = 0.000), comprising a direct effect (β = 0.293, 95% CI [0.140–0.438], *P* = 0.000) and an indirect effect mediated by ESE (β = 0.085, 95% CI [0.039–0.146], *P* = 0.000). ESE also exerted a significant direct effect on MVPA (β = 0.35, 95% CI [0.229–0.450], *P* < 0.001). The model explained 26.3% of the variance in MVPA.

**Conclusion:**

This study highlights that both PS and ESE are associated with MVPA in children and adolescents with VI, with ESE acting partially mediating this relationship.

## Introduction

Physical activity (PA) is widely recognized as essential for the physical and psychological well-being of children and adolescents with disabilities. Regular PA enhances muscle and bone strength, improves aerobic capacity, and promotes mental health, cognitive function, overall well-being, and quality of life (Dubon, Tow & Rabatin, 2023; Prieto et al., 2024). Cross-national studies have shown that children and adolescents with visual impairment (VI) tends to adopt more sedentary lifestyles than their sighted peers, increasing their risk of obesity and other health problems during both childhood and adulthood (Aslan, Calik & Kitis, 2012; Augestad & Jiang, 2015; Guthold et al., 2020; Haegele & Porretta, 2015; Kozub & Oh, 2004). In response to these concerns, the World Health Organization (WHO) issued its first PA and sedentary behavior guidelines for people with disabilities in 2020, recommending that children and adolescents with disabilities aged 5–17 years should accumulate an average of at least 60 min of moderate-to-vigorous physical activity (MVPA) per day throughout the week (World Health Organization, 2020). Despite these recommendations, evidence consistently indicates that this population fails to meet these guidelines (Brian et al., 2019; Dou et al., 2025), with longitudinal studies further demonstrating a progressive decline in MVPA throughout adolescence (Brian et al., 2019; Gür et al., 2020; Haegele, Zhu & Kirk, 2018b). Empirical studies further indicate that children and adolescents with VI exhibit the lowest PA levels among all disability groups and are the least likely to engage in PA, a trend that has drawn increasing attention from public health authorities worldwide (Longmuir & Bar-Or, 2010). Children and adolescents who participate in MVPA are more likely to maintain these healthy habits into adulthood (Huotari et al., 2011). Although disability may attenuate some health benefits typically associated with increased PA (Carty et al., 2021), enhancing MVPA among children and adolescents with VI remains a critical public health priority, conferring substantial benefits for cardiovascular, metabolic, and mental health.

MVPA is defined as activity with an intensity equivalent to 3–8.7 metabolic equivalents (Garber et al., 2011). Engagement in MVPA occurs within specific environmental contexts, where personal behaviors interact with the surrounding environments and society. The family is one of the earliest and primary social environments, and a crucial component of which is parental support (PS). PS encompasses the various mechanisms through which parents influence their children’s behavioral development (Beets, Cardinal & Alderman, 2010) and has been identified as a key determinant of PA participation among adolescents (Yao & Rhodes, 2015). This influence is consistent with Social Cognitive Theory, which posits that personal, behavioral, and environmental factors interactively determine PA behaviors (Bandura, 2004). From a socio-ecological perspective (Welk, 1999), Youth Physical Activity Promotion Model similarly identifies family and social environmental support as reinforcing factors for youth PA. Similarly, van der Ploeg et al. (2004), based on the ICF disability model, proposed a PA model for individuals with disabilities, categorizing relevant factors of PA into environmental and personal dimensions, while emphasizing the crucial role of the family environment and social support. Collectively, these theoretical frameworks underscore the importance of social support in behavioral change, with PS representing a major source of such support for adolescents regardless of whether they are typically developing or have disabilities (Bandura, 2001). Studies have shown that the greater the amount of social support children and adolescents receive, the higher the level of PA in which they are likely to engage (Mendonca et al., 2014).

Beyond environmental factors, individual psychological characteristics are important determinants of PA. Understanding the psychological mechanisms behind these behaviors is critical for implementing effective interventions. Exercise self-efficacy (ESE) defined as an individual’s confidence in overcoming barriers to maintain regular PA (Voskuil & Robbins, 2015) has been consistently associated with PA behavior. Social Cognitive Theory posits that strong social support enhances self-efficacy to overcome barriers to PA (Bandura, 2001), and self-efficacy serves as both a core variable in behavioral change and a primary predictor of PA levels. Research has found that PS significantly enhances children’s self-efficacy, thereby stimulating their motivation to exercise and increasing PA levels (Trost et al., 2003). Furthermore, parents’ active involvement and instrumental social support can influence children’s PA indirectly through their sports-related cognitions and self-efficacy (Peterson et al., 2013). Haegele, Kirk & Zhu (2018a) demonstrated that ESE is independently and positively associated with MVPA time among U.S. adults with VI, and a study of Japanese adults with VI also reported a similar positive correlation (Tatsuta et al., 2022). Evidence from Chinese adolescents with intellectual disabilities also supports this relationship, showing a significant positive correlation between ESE and weekend MVPA (Chao et al., 2016).

Various studies have explored the mechanisms through which PS promotes PA in children and adolescents. While some research has examined parental role modeling as an explanatory mechanism (Moore et al., 1991), this perspective alone is insufficient to fully elucidate the psychological factors underlying children’s PA engagement. In studies involving typically developing children and adolescents, the influence of parental social support on PA has been shown to be mediated by several socio-psychological variables, such as children’s self-efficacy and self-esteem (Ornelas, Perreira & Ayala, 2007). While the mediating role of self-efficacy is well established in general adolescent populations (Trost et al., 2003), this mechanism may be particularly pronounced among children and adolescents with VI. These individuals face compounded environmental and psychosocial barriers to PA (*e.g*., inaccessible facilities, safety concerns, and exclusion from mainstream sports), which can severely undermine efficacy beliefs (Dixon-Ibarra & Driver, 2013).

Therefore, the present study aims to examine the relationships among PS, ESE, and MVPA in Chinese children and adolescents with VI, and to test potential mediating role of ESE in this context. The hypothesized model is depicted in Fig. 1. The hypotheses are as follows:

(H1) PS, ESE, and MVPA are positively correlated;
(H2) PS and ESE significantly and positively predict MVPA;
(H3) ESE plays a mediating role in the relationship between PS and MVPA.

**Figure 1:**
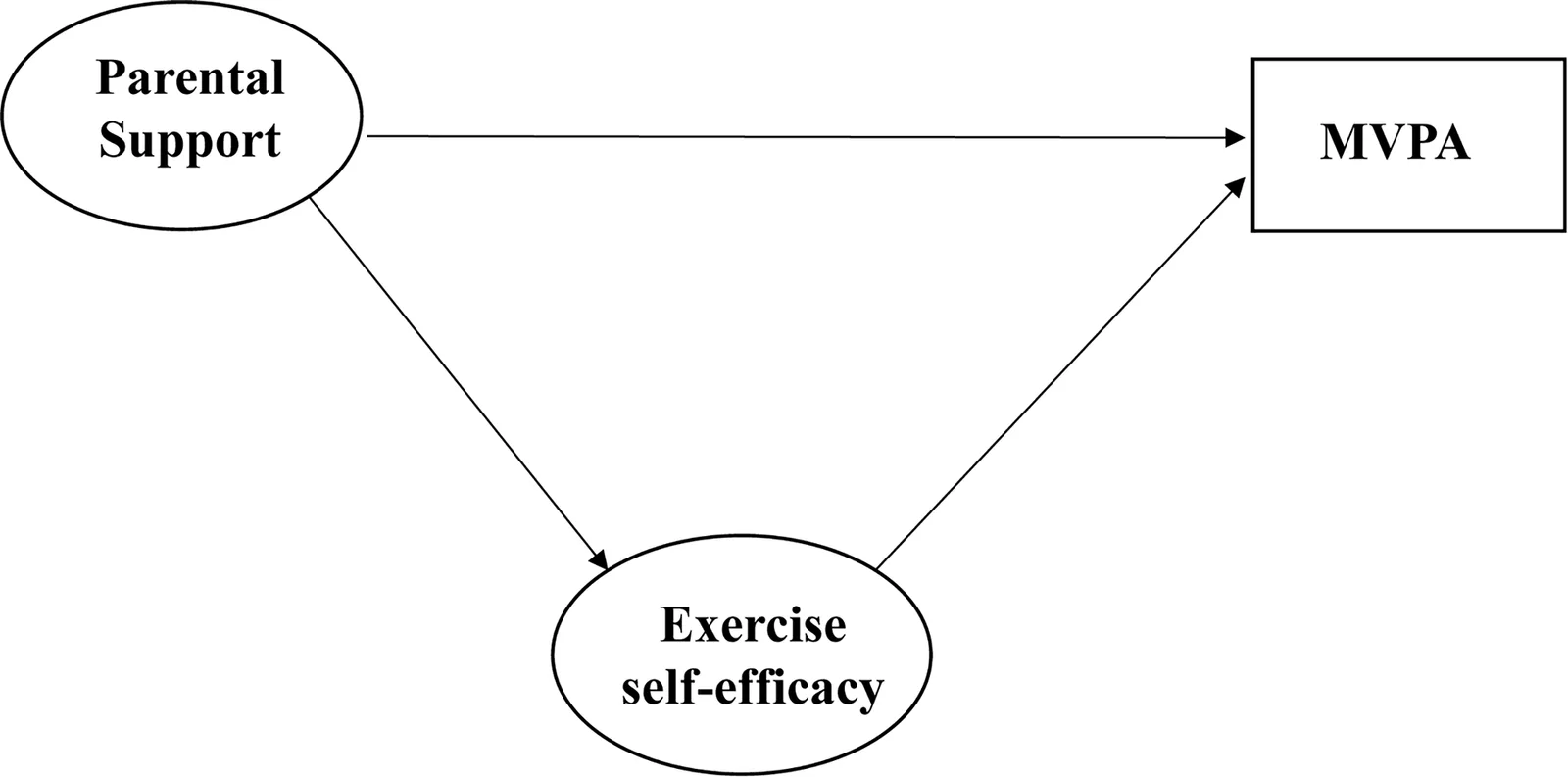
A hypothetical model of the relationship.

## Materials and methods

### Participants

A total of 340 children and adolescents with congenital VI aged 7–18 years were initially recruited from six special education schools in the East China. The participants, who included individuals with total blindness and low vision, were recruited from three schools in Jiangsu Province, two in Anhui Province, and one in Shanghai City. The diagnosis of VI was based on the national standard of the People’s Republic of China, “Classification and Grading of Disabilities” (AQSIQ, 2011). The sample size of the present study is relatively limited, primarily due to the inherent challenges of recruiting a large cohort of children and adolescents with VI, which is a low-incidence population. Six participants were excluded due to instrument failure resulting in missing PA data. Another 18 participants were excluded due to invalid accelerometer data, defined as wearing the device for fewer than 2 weekdays and 1 weekend day, or for less than 8 h per day. Additionally, 10 participants were excluded due to invalid questionnaire data. Ultimately, 306 valid datasets were included in the analysis, comprising 163 boys and 143 girls.

### Data collection procedures

Data collection was conducted at the participating schools during school hours from September to December 2024. Clear explanations of key concepts related to the outcomes, including PA, ESE, and PS were explained to the participants before they completed the survey. Each child completed a two-page survey that included questions about their PS for PA, and ESE, and other demographic information. It took approximately 15 min for each participant to complete. This study was approved by the research Ethics Committee of Nanjing Sport University (No. RT-2022-10), and data were collected by trained research assistants and teachers. All participants were enrolled voluntarily, and written informed consent was obtained from their parents or guardians.

### Measures

#### Assessment of physical activity

GT3X+ accelerometer (ActiGraph, Pensacola, FL, USA) was used to measure MVPA and sedentary time. This device has good reliability and validity in children and adolescents with VI (Kozub, Oh & Rider, 2005). Before the test, students’ information was recorded. The accelerometers were attached to the right hip of each participant by researchers and school staff, who also provided instructions on proper wear and care. Participants were required to wear the devices continuously for 7 days, including 5 weekdays and 2 weekend days, and to remove them only during bathing and swimming. Physical education (PE) teachers and class teachers supervised students’ wear during school days, while parents were responsible for supervision on weekends. After the 7-day period, the accelerometers were collected by the PE teachers and handed over to the research team. The entire data collection process was coordinated by two research team members, three PE teachers, and each class director.

The raw data were imported into the accelerometer software for processing and a 15-s epoch was used to record the data. Valid accelerometer data were defined as a minimum of 8 h of wear time per day, with at least 2 valid weekdays and 1 valid weekend day required for inclusion. In the data analysis, a continuous zero count of ≥60 min was adopted as the criterion for determining non-wear time. The cutoff points formulated by Evenson et al. (2008) were applied, as they have been shown to be suitable for school-aged children (Trost et al., 2011) and have been utilized in prior studies among children and adolescents with disabilities, including VI (Sit et al., 2019, 2017). Sedentary time was defined as <100 counts per minute (cpm), and MVPA is defined as ≥2,296 cpm, including moderate physical activity (MPA; 2,296–4,011 cpm), and vigorous physical activity (VPA; ≥4,012 cpm). Daily MVPA time was calculated, and the percentage of MVPA time (%MVPA) relative to total wear time was derived.

#### Parental support for physical activity

PS for children’s and adolescents’ PA was assessed using the Parental Support Scale, which was originally developed by Prochaska, Rodgers & Sallis (2002) and subsequently translated, culturally adapted, and validated for use in China by Liu et al. (2017). The items are as follows:

PS1, parental PA encouragement: “Do your parents frequently encourage you to engage in physical activity and exercise?”
PS2, parental accompanying: “Do your parents often accompany you in physical activity and exercise?”
PS3, parental involvement: “Do your parents regularly engage in physical activity and exercise with you?”
PS4, parental financial support: “Do your parents provide transportation and financial support for your physical activity and exercise participation?”
PS5, parental role modeling: “Do your parents motivate you to engage in physical activity and exercise by setting an example through their own behavior?”

Responses were recorded on a 5-point Likert scale ranging from 1 (“never”) to 5 (“always”). The total score of all items was calculated, with higher scores indicating stronger PS. The Parental Support Scale has been previously validated in the VI adolescent population (Greguol, Gobbi & Carraro, 2015). In the present sample, this scale demonstrated good internal consistency, with a Cronbach’s alpha coefficient of 0.856. Overall, this questionnaire has demonstrated feasibility, acceptable reliability, and validity in studies involving children and adolescents with VI (Greguol, Gobbi & Carraro, 2015).

#### Exercise self-efficacy

The ESE Scale, developed by Pirasteh et al. (2008), was used to assess self-efficacy in PA. The questionnaire comprises six items that evaluate confidence in maintaining regular exercise under various challenging conditions:

ESE1: Engaging in physical activity even when feeling sad or stressed;
ESE 2: Setting aside time for physical activity on most days;
ESE 3: Engaging in physical activity even when family members want me to do something else;
ESE 4: Confidence in persist with exercise when needing to wake up early;
ESE 5: Confidence in persisting with exercise when faced with a heavy academic workload;
ESE 6: Confidence in persisting with exercise during unfavorable weather conditions.

Each item uses a 5-point Likert scale reflecting different degrees of confidence in exercising regularly, ranging from 1 (“not confident”) to 5 (“very confident”). The total score of all items was calculated as the outcome measure. In the present sample, this scale demonstrated good internal consistency, with a Cronbach’s alpha coefficient of 0.879. This scale has demonstrated a test–retest reliability of 0.68 among children and adolescents (Pirasteh et al., 2008) and a good applicability among Chinese adolescents with disabilities (Chao et al., 2016).

### Data analysis

Data processing and analysis were performed using SPSS 24.0. The Shapiro-Wilk test was applied to assess the normality of all continuous variables. Variables that conformed to a normal distribution were reported as mean ± standard deviation $(\bar{x} \pm s)$. Gender and age differences in all outcomes were examined using independent-samples *t*-tests. The correlations among PA, ESE, and PS were examined using Pearson’s partial correlation coefficients, controlling for gender and age, with statistical significance set at *P* < 0.05.

Structural equation modeling (SEM) was performed with AMOS 26.0 to evaluate the hypothesized model and to examine the potential mediating role of ESE in the relationship between PS and MVPA. The final analytical sample included 306 participants. As the sample size exceeded 10 times the number of free parameters, the sample was considered sufficient to meet the stability requirements for model parameter estimation. To test the significance of the indirect effect, a nonparametric bias-corrected (BC) percentile bootstrap method was employed with 5,000 resamples drawn with replacement from the full analytical sample. Specifically, the analysis generated 95% confidence intervals (CIs) using the BC percentile bootstrap method. The direct and indirect effects were considered statistically significant if the 95% CI did not include zero. The effect size for the mediation effect was calculated as the ratio of the indirect effect to the total effect. Model fit was assessed using multiple indices, including the chi-square statistic (*χ*^2^) with degrees of freedom (*df*), the *χ*^2^*/df* ratio, goodness-of-fit index (GFI), the adjusted goodness-of-fit index (AGFI), and the root-mean-square error of approximation (RMSEA). Adequate model fit was indicated by the following criteria: *χ*^2^*/df* between 1 and 3, RMSEA <0.08, and incremental indices, including NFI, IFI, CFI, RFI, TLI, and GFI, all ≥0.90.

Common method variance (CMV) was assessed using Harman’s single-factor test (Hairman, 2009). An exploratory factor analysis that included all survey items yielded two factors with eigenvalues greater than 1. The first factor explained 38.29% of the total variance, which was well below the 40% threshold, indicating that CMV did not pose a meaningful threat to the validity of the findings (Wu, Zhao & Zhao, 2022).

## Results

### MVPA, ESE, and PS in children and adolescents with VI

Table 1 presents the activity patterns of participants with VI. Overall, this population spent 80.38% of their time in sedentary behavior and only 5.11% in MVPA. Participants aged 7–12 years accumulated a significantly higher percentage of MVPA and a lower percentage of sedentary time than those aged 13–18 years (both *P* < 0.05). No significant differences in PS scores were observed across sex or age groups (*P* > 0.05). Additionally, ESE was significantly lower in adolescents than in children (*P* < 0.01).

**Table 1:** Descriptive statistics (M ± SD) and gender-/age-related differences in %MVPA, exercise self-efficacy and perceived parental support.

		*n*	Wearing time (min)	%MVPA	%ST	Parental support	Exercise self-efficacy
		306	719.62 ± 128.98	5.11 ± 2.37	80.38 ± 6.11	15.78 ± 4.06	18.92 ± 5.32
Gender	Boys	163	707.80 ± 129.38	5.34 ± 2.48	79.62 ± 6.54	15.83 ± 4.15	19.04 ± 5.28
	Girls	143	733.10 ± 127.63	4.86 ± 2.21	81.25 ± 5.47	15.73 ± 3.97	18.80 ± 5.38
	*F*		0.024	3.513	0.256	0.653	0.116
	*P*		0.087	0.080	0.604	0.840	0.695
Age	Children	134	713.30 ± 126.20	6.01 ± 2.69	79.55 ± 6.27	16.25 ± 4.22	19.82 ± 4.96
	Adolescent	172	724.55 ± 131.25	4.41 ± 1.80	81.03 ± 5.92	15.42 ± 3.90	18.23 ± 5.49
	*F*		0.572	9.167	4.480	2.536	6.901
	*P*		0.450	0.001	0.035	0.07	0.009

**Note:**

M, mean; SD, standard deviation; %MVPA, percentage of moderate-to-vigorous physical activity in total wear time; %ST, percentage of sedentary time in total wear time.

### Associations between MVPA, ESE and PS

Table 2 shows that PS and its subdimensions were positively correlated with %MVPA (*r* = 0.173–0.306, *P* < 0.05) after adjusting for age, gender, and daily valid wear time. Among children and adolescents with VI, PS was positively associated with ESE (*r* = 0.202, *P* < 0.05). PS and its dimensions showed significant positive correlations with ESE (*r* = 0.113–0.211, *P* < 0.05). Moreover, ESE scores were strongly and positively associated with %MVPA (*r* = 0.385, *P* < 0.05).

**Table 2:** Correlation analysis results.

	MVPA	PS	ESE	PS1	PS2	PS3	PS4	PS5
MVPA	–							
PS	0.306*	***0.740***						
ESE	0.385*	0.202*	***0.733***					
PS1	0.173*	0.777*	0.113*	–				
PS2	0.303*	0.799*	0.160*	0.614*	–			
PS3	0.204*	0.763*	0.140*	0.439*	0.446*	–		
PS4	0.279*	0.836*	0.179*	0.579*	0.610*	0.557*	–	
PS5	0.261*	0.801*	0.211*	0.466*	0.530*	0.581*	0.584*	–

**Notes:**

* *P* < 0.05.

Diagonal elements (in bold italics) represent Square roots of average variance extracted (AVE) values; The off-diagonal elements represent the inter-construct Pearson correlation coefficients. MVPA, moderate-to-vigorous physical activity; PS1, parental PA encouragement; PS2, parental accompanying; PS3, parental involvement; PS4, parental financial support; PS5, parental role modeling; ESE, exercise self-efficacy.

### Measurement model and structural model path analysis

The measurement model exhibited good reliability and validity. All standardized factor loadings were statistically significant (*P* < 0.01) and surpassed the conventional threshold of 0.5, with the majority exceeding the ideal level of 0.7 (Table 3). The squared multiple correlations (SMC) for each indicator, representing the proportion of variance explained by the latent construct, ranged from 0.43 to 0.67. All values exceeded the recommended threshold of 0.36 (Hair et al., 2020), and most were above 0.5, indicating good to excellent indicator reliability. Composite reliability (CR) exceeded the recommended 0.7, demonstrating good construct reliability for all latent variables. Finally, the average variance extracted (AVE) values for all constructs were above the minimum value of 0.5, suggesting good convergent validity. The measurement models of PS, ESE, demonstrated good model fit, with *χ*^2^*/df* = 1.932 for PS and χ^2^/*df* = 1.650 for ESE. For all models, CFI, GFI, AGFI, TLI, and IFI were >0.90, and RMSEA was <0.08. In addition, correlations among constructs were lower than the square roots of the AVE values for each latent variable, indicating that the discriminant validity was achieved.

**Table 3:** Measurement model: indicators and loadings of directly measured variables on constructs.

Latent construct	Indicator	Standardized loadings	*P*-value	CR	AVE
Parental support	PS1	0.714	<0.001	0.879	0.548
	PS2	0.769	<0.001		
	PS3	0.654	<0.001		
	PS4	0.820	<0.001		
	PS5	0.729	<0.001		
Exercise self-efficacy	ESE1	0.710	<0.001	0.851	0.537
	ESE2	0.746	<0.001		
	ESE3	0.785	<0.001		
	ESE4	0.723	<0.001		
	ESE5	0.753	<0.001		
	ESE6	0.721	<0.001		

**Note:**

CR, composite reliability; AVE, average variance extracted, CR values above 0.7 and AVE values above 0.5 indicate good reliability and convergent validity, respectively.

The hypothesized structural relationships among ESE, PS, and %MVPA were examined using SEM. As shown in Fig. 2, the model demonstrated a good fit to the data. The fit indices indicated acceptable overall fit: *χ*^2^*/df* = 2.406, GFI = 0.936, AGFI = 0.904, NFI = 0.924, CFI = 0.954, and RMSEA= 0.068. The partial mediation structural model accounted for 26.3% of the variance in MVPA. Path tests of the structural equation modeling showed significant path coefficients (*P* < 0.01) for the direct effects of PS → ESE, PS → %MVPA, and ESE → %MVPA (Fig. 2). The direct effects of PS on PA and ESE were 0.29 and 0.24, respectively, and ESE had a direct effect of 0.35 on %MVPA.

**Figure 2:**
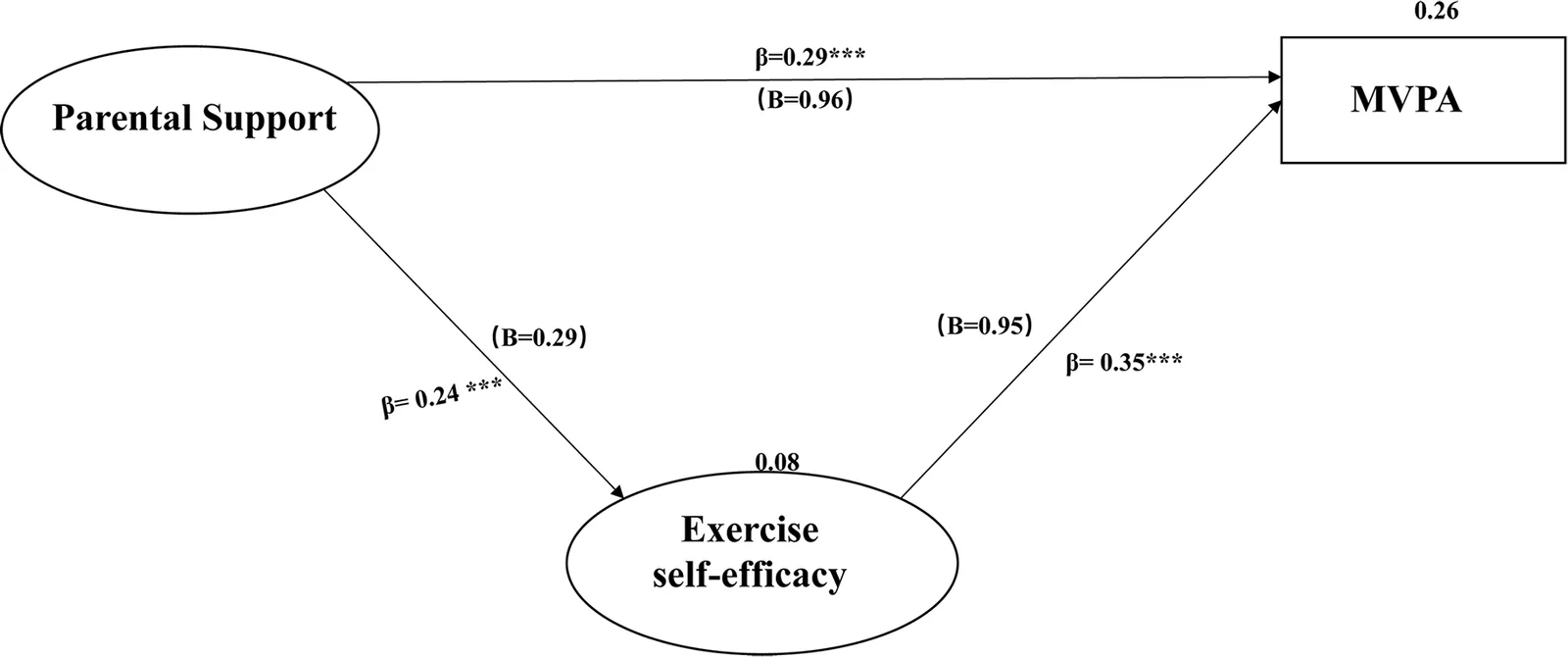
The results of structural equation modeling analysis. Standardized coefficients (β) are presented above each path, rounded to two decimal places. Nonstandardized coefficients (B) are shown below each path in parentheses. ***P < 0.001.

### The mediating role of ESE

The BC percentile bootstrap method was used to test the pathway PS → ESE → %MVPA by repeatedly resampling the original data to generate 5,000 bootstrap samples. The results are shown in Table 4. The total effect of PS on %MVPA was 0.379 (95% CI [0.226–0.523], *P* = 0.000), with the CI excluding 0, indicating a significant effect. The direct effect of PS on %MVPA was 0.293 (95% CI [0.140–0.438], *P* = 0.000), with the CI excluding 0, indicating a significant effect. The indirect effect of PS on %MVPA was 0.085 (95% CI [0.039–0.146], *P* = 0.000), with the CI excluding 0, indicating a significant mediating effect.

**Table 4:** The mediating effect of exercise self-efficacy on parental support and %MVPA.

Path relationship		Std. estimates	SE	Bias 95% CI		Percentile 95% CI	
				Lower	Upper	Lower	Upper
PS → ESE → %MVPA	Total effect	0.379	0.076	0.226	0.523	0.230	0.527
	Direct effect	0.293	0.077	0.140	0.438	0.144	0.442
	Indirect effect	0.085	0.027	0.039	0.146	0.036	0.141

**Note:**

% MVPA, percentage of moderate-to-vigorous physical activity in total wear time; PS, parental support; ESE, exercise self-efficacy.

## Discussion

Participants in this sample spent 80.38% of wear time in sedentary behavior and only 5.11% (43.62 min/day) in MVPA. This proportion of MVPA is substantially lower than the 16.8% reported among typically-sighted adolescents (Hong et al., 2020), highlighting the predominantly inactive lifestyle of this population. These findings indicate that children and adolescents with VI in this study engaged in low levels of PA and spent most of their time in sedentary behavior, consistent with previous international studies (Aslan, Calik & Kitis, 2012; Gür et al., 2020). Additionally, significant age-related differences in %MVPA were observed, with the 7–12-year-old group exhibiting significantly higher %MVPA than the 13–18-year-old group. This finding aligns with previous literature, reporting a significant negative correlation between age and PA levels (Kozub & Oh, 2004). As children transition into adolescence, the duration of MVPA tends to decline, while sedentary time correspondingly increases. This pattern may be attributed to multiple factors, including diminished motivation for PA and heightened concerns about sports-related injuries (Bloemen et al., 2015). The present study also identified significant age-related differences in ESE. Compared with adolescents aged 13–18 years, children with VI aged 7–12 years showed significantly greater confidence in overcoming barriers to PA participation. This finding has important practical implications: interventions to promote PA among adolescents with VI should prioritize strategies to enhance ESE, with tailored approaches developed specifically for older adolescents to address their unique needs.

It is well established that PS plays an important role in the development of children’s healthy lifestyle behaviors. Doggui, Gallant & Bélanger (2021) demonstrated that PS for PA predicts adolescents’ MVPA over a 5-year period, underscoring its long-term impact on PA behaviors. Similarly, Pluta et al. (2020) found that adolescents’ PA behavior is significantly associated with their perceptions of PS, highlighting the importance of parental encouragement and involvement in promoting healthy behaviors. However, research examining the relationship between parental support and PA in Chinese adolescents with disabilities, particularly those with VI, remains scarce. The first aim of this study was therefore to test a conceptual model linking PS and PA among children and adolescents with VI.

The present study revealed a significant positive correlation between PS and %MVPA in children and adolescents with VI, consistent with previous research demonstrating a strong association between PS and PA in general populations (Liu et al., 2017) and among adolescents with VI in particular (Greguol, Gobbi & Carraro, 2015; Robinson & Lieberman, 2007). Notably, previous studies have rarely examined distinct dimensions of PS in relation to MVPA. The present findings demonstrate that all domains of PS are positively associated with %MVPA among children and adolescents with VI. Failure to satisfy their fundamental needs may severely constrain their capacity to overcome the multiple barriers to engaging in PA (Doggui, Gallant & Bélanger, 2021). According to Voskuil & Robbins (2015), the effectiveness of PS in promoting PA among children depends on the child’s perception of its adequacy. These findings align with Liu et al. (2017), who reported that five parental support dimensions were positively linked to %MVPA in a representative sample of typically developing adolescents. This suggests that children and adolescents with VI may depend more heavily on multidimensional PS to initiate and sustain MVPA than their typically developing counterparts. This view is supported by Gillison et al. (2017), who found that PS is associated with higher levels of MVPA. International research has further identified parental encouragement, involvement, and support (Columna et al., 2017; Perkins et al., 2013), as well as the provision of transportation and safety (Ayvazoglu, Oh & Kozub, 2006), as key factors in successfully promoting PA among children and adolescents with VI. Similarly, a study of children with autism found that the amount of time parents spend encouraging PA significantly predicts their children’s MVPA participation (Lee, Healy & Haegele, 2022).

The second objective of this study was to elucidate the mechanistic pathways through which PS and ESE predict %MVPA among children and adolescents with VI. Although prior work, predominantly conducted in Western countries, has linked both PS and ESE to PA across age and sex groups with disabilities, empirical evidence from China remains scarce, particularly for this population. To mitigate the systematic error inherent in self-reported activity data, MVPA was obsessed objectively in this study, thereby offering a methodologically robust extension of the existing literature. The present findings indicate that PS exerts significant direct effects on both ESE and MVPA among children and adolescents with VI. ESE, in turn, was found to be independently and positively associated with MVPA, and mediation analysis suggested that it may account for part of the association between PS and MVPA. These results align with Trost et al. (2003), who demonstrate that parental support is a salient correlate of adolescent PA and may be associated with PA engagement both directly and indirectly through the enhancement of ESE.

The mediating role of ESE in the relationship between social support and PA has been well-documented in previous literature (Gillison et al., 2017; Trost et al., 2003). Prior research has used self-reported PA to examine the relationships among PS, ESE, and adolescent PA (Peterson et al., 2013), whereas the present study used accelerometer-based assessments. This study extends existing evidence to children and adolescents with VI. The results demonstrate that PS exerts an indirect effect on %MVPA through ESE, which acts as a partial mediator. Specifically, higher levels of PS were found to be associated with stronger ESE, which in turn was related to higher levels of MVPA. The mediating effect analysis showed that ESE accounted for 22.43% of the total effect of PS on %MVPA, indicating that, in addition to strengthening PS, enhancing adolescents’ ESE is essential for improving their MVPA.

PS emerged as a significant predictor of MVPA in the present study. However, the model explained only 26.3% of the variance, indicating that a substantial proportion of MVPA engagement among children and adolescents with VI is attributable to other factors, such as exercise motivation and beliefs (Guo, 2022). Other important factors may include environmental accessibility, such as inclusive recreational facilities; peer influence through modeling and social support; and school-based variables, such as adapted PE curricula and extracurricular sports (Martin Ginis et al., 2016). Future research would benefit from a more comprehensive, multilevel framework integrating family, peer, school, and environmental determinants to better capture the multifaceted nature of PA in this population. These findings should also be interpreted within China’s cultural context, where PS for PA among adolescents with VI may differ from Western patterns. China’s segregated special-school system also creates a relatively homogeneous peer environment with adapted PE, potentially amplifying school-based ESE while diminishing the unique role of parental support (Qi, Xu & Shao, 2020). Although the broad mediation pathway appears consistent across cultures, its underlying mechanisms likely differ in both magnitude and meaning from Western inclusive-education contexts. This may be particularly true for the balance between direct parental facilitation and efficacy-mediated motivation.

During puberty, a critical developmental stage, PS serves as a favorable external environmental factor that can enhance the confidence of children and adolescents with VI in overcoming barriers, fostering their motivation to engage in exercise, and thereby provide a reliable internal drive for PA participation, ultimately promoting MVPA. These findings further validate the crucial mediating role of ESE between the interpersonal and individual levels of the socio-ecological model. Consistent with Social Cognitive Theory (Bandura, 2001), social-support networks enhance ESE for overcoming barriers to PA, thereby fostering sustained engagement. Among individuals with disabilities, this efficacy belief is especially critical: by transforming perceptions of functional limitation into appraisals of personal competence, it strengthens intrinsic motivation and, consequently, participation in PA (Dixon-Ibarra & Driver, 2013).

## Strengths and limitations

This study was characterized by several limitations. First, the sample was drawn from six special education schools in East China, which may not fully represent the diversity of children and adolescents with VI. Because the sample was recruited from a specific geographic and cultural context, the participating schools may differ from others in terms of PE curricula and extracurricular activity arrangements, which could influence the observed relationships between PS and PA. Additionally, the convenience sampling approach may have introduced systematic differences in family socioeconomic status and VI severity compared to the broader population of children and adolescents with VI. Therefore, the findings may be most applicable to school-aged children and adolescents with VI who have similar demographic and contextual characteristics, rather than to all children with VI. Future studies should expand recruitment to include multiple regions and school types. Second, the cross-sectional design precludes causal inferences regarding PS and PA; longitudinal or experimental approaches, such as assessing PA changes following PS interventions, are needed to establish temporal relationships. Finally, by focusing primarily on PS and ESE, other potential confounders, such as school policies and, peer influence, may have been overlooked. Future research should adopt a socioecological framework to examine multilevel determinants or use SEM to analyze complex pathways.

## Conclusion

The findings demonstrate that PS in children and adolescents with VI is significantly and positively correlated with both ESE and MVPA. Notably, ESE partially mediates the relationship between PS and MVPA. Future interventions should consider the creation of favorable PS environments for adolescents with VI to increase their ESE in overcoming PA barriers as an effective pathway to promote MVPA participation in this population.

## Supplemental Information

10.7717/peerj.21871/supp-1 Supplemental Information 1 Raw data.

10.7717/peerj.21871/supp-2 Supplemental Information 2 Codebook.

10.7717/peerj.21871/supp-3 Supplemental Information 3 STROBE Checklist.

10.7717/peerj.21871/supp-4 Supplemental Information 4 Flowchart.

10.7717/peerj.21871/supp-5 Supplemental Information 5 Articles including Exercise Self-Efficacy (ESE) Scale.

10.7717/peerj.21871/supp-6 Supplemental Information 6 Chinese scale including Exercise Self-Efficacy(ESE) Scale.

10.7717/peerj.21871/supp-7 Supplemental Information 7 Exercise Self-Efficacy(ESE) Scale.

10.7717/peerj.21871/supp-8 Supplemental Information 8 Articles including Exercise Self-Efficacy (ESE) Scale by Pirasteh et al. (2008).

10.7717/peerj.21871/supp-9 Supplemental Information 9 Association of Parent and Peer Support with Adolescent Physical Activity.

10.7717/peerj.21871/supp-10 Supplemental Information 10 Development and validation of psychosocial determinants measures of physical activity among Iranian adolescent girls.

## Additional Information and Declarations

### Data Availability

The following information was supplied regarding data availability:

The raw data is available in the Supplemental Files.

## References

[ref-1] AQSIQ (2011). Classification and grading of disabilities: GB/T26341-2010.

[ref-2] Aslan UB, Calik BB, Kitis A (2012). The effect of gender and level of vision on the physical activity level of children and adolescents with visual impairment. Research in Developmental Disabilities.

[ref-3] Augestad LB, Jiang L (2015). Physical activity, physical fitness, and body composition among children and young adults with visual impairments: a systematic review. British Journal of Visual Impairment.

[ref-4] Ayvazoglu NR, Oh HK, Kozub FM (2006). Explaining physical activity in children with visual impairments: a family systems approach. Exceptional Children.

[ref-5] Bandura A (2001). Social cognitive theory: an agentic perspective. Annual Review of Psychology.

[ref-6] Bandura A (2004). Health promotion by social cognitive means. Health Education & Behavior.

[ref-7] Beets MW, Cardinal BJ, Alderman BL (2010). Parental social support and the physical activity-related behaviors of youth: a review. Health Education & Behavior.

[ref-8] Bloemen MAT, Backx FJG, Takken T, Wittink H, Benner J, Mollema J, De Groot JF (2015). Factors associated with physical activity in children and adolescents with a physical disability: a systematic review. Developmental Medicine & Child Neurology.

[ref-9] Brian A, Pennell A, Haibach-Beach P, Foley J, Taunton S, Lieberman LJ (2019). Correlates of physical activity among children with visual impairments. Disability and Health Journal.

[ref-10] Carty C, van der Ploeg HP, Biddle SJ, Bull F, Willumsen J, Lee L, Kamenov K, Milton K (2021). The first global physical activity and sedentary behavior guidelines for people living with disability. Journal of Physical Activity and Health.

[ref-11] Chao W, Gang HE, Jianzhong LI, Yan Z (2016). Physical activity levels and exercise self-efficacy in adolescents with disability: a preliminary study using accelerometry. Journal of Capital University of Physical Education and Sports.

[ref-12] Columna L, Rocco Dillon S, Norris ML, Dolphin M, McCabe L (2017). Parents’ perceptions of physical activity experiences for their families and children with visual impairments. British Journal of Visual Impairment.

[ref-13] Dixon-Ibarra A, Driver S (2013). The role of self-efficacy in physical activity participation for persons with disabilities. Palaestra.

[ref-14] Doggui R, Gallant F, Bélanger M (2021). Parental control and support for physical activity predict adolescents’ moderate to vigorous physical activity over five years. International Journal of Behavioral Nutrition and Physical Activity.

[ref-15] Dou L, Chen HW, Xia YM, Lu EJ (2025). Seasonal variations in moderate-to-vigorous physical activity among children and adolescents with visual impairment during different segments of the school day. Frontiers in Public Health.

[ref-16] Dubon ME, Tow S, Rabatin AE (2023). Physical activity and sports participation among children and adolescents with disabilities. Pediatric Clinics.

[ref-17] Evenson KR, Catellier DJ, Gill K, Ondrak KS, McMurray RG (2008). Calibration of two objective measures of physical activity for children. Journal of Sports Sciences.

[ref-18] Garber CE, Blissmer B, Deschenes MR, Franklin BA, Lamonte MJ, Lee IM, Nieman DC, Swain DP, Med ACS (2011). Quantity and quality of exercise for developing and maintaining cardiorespiratory, musculoskeletal, and neuromotor fitness in apparently healthy adults: guidance for prescribing exercise. Medicine & Science in Sports & Exercise.

[ref-19] Gillison FB, Standage M, Cumming SP, Zakrzewski-Fruer J, Rouse PC, Katzmarzyk PT (2017). Does parental support moderate the effect of children’s motivation and self-efficacy on physical activity and sedentary behaviour?. Psychology of Sport and Exercise.

[ref-20] Greguol M, Gobbi E, Carraro A (2015). Physical activity practice among children and adolescents with visual impairment–influence of parental support and perceived barriers. Disability and Rehabilitation.

[ref-21] Guo S (2022). The relationship between parental support and adolescents' exercise adherence: the chain mediation effects of exercise motivation and self-efficacy.

[ref-22] Guthold R, Stevens GA, Riley LM, Bull FC (2020). Global trends in insufficient physical activity among adolescents: a pooled analysis of 298 population-based surveys with 1· 6 million participants. The Lancet Child & Adolescent Health.

[ref-23] Gür K, Beyhan A, Aktan I, Akbulut E, Sezer M, Elik E, Akc T (2020). Physical activity levels, enjoyment, and perceptions of barriers to physical activity of adolescents with visual impairments in Turkey. Journal of Visual Impairment & Blindness.

[ref-24] Haegele JA, Kirk TN, Zhu XH (2018a). Self-efficacy and physical activity among adults with visual impairments. Disability and Health Journal.

[ref-25] Haegele JA, Porretta D (2015). Physical activity and school-age individuals with visual impairments: a literature review. Adapted Physical Activity Quarterly.

[ref-26] Haegele JA, Zhu XH, Kirk TN (2018b). Weekday physical activity and health-related fitness of youths with visual impairments and those with autism spectrum disorder and visual impairments. Journal of Visual Impairment & Blindness.

[ref-27] Hair JF, Black WC, Babin BJ, Anderson RE (2020). Multivariate data analysis.

[ref-28] Hairman JF (2009). Multivariate data analysis.

[ref-29] Hong JT, Chen ST, Tang Y, Cao ZB (2020). Associations between different types of parental support and physical activity among children and adolescents.

[ref-30] Huotari P, Nupponen H, Mikkelsson L, Laakso L, Kujala U (2011). Adolescent physical fitness and activity as predictors of adulthood activity. Journal of Sports Sciences.

[ref-31] Kozub FM, Oh H-K (2004). An exploratory study of physical activity levels in children and adolescents with visual impairments. Clinical Kinesiology.

[ref-32] Kozub FM, Oh HK, Rider RA (2005). RT3 accelerometer accuracy in estimating short term physical activity in individuals with visual impairments. Adapted Physical Activity Quarterly.

[ref-33] Lee J, Healy S, Haegele JA (2022). Environmental and social determinants of leisure-time physical activity in children with autism spectrum disorder. Disability and Health Journal.

[ref-34] Liu Y, Zhang YJ, Chen ST, Zhang JL, Guo ZC, Chen PJ (2017). Associations between parental support for physical activity and moderate-to-vigorous physical activity among Chinese school children: a cross-sectional study. Journal of Sport and Health Science.

[ref-35] Longmuir PE, Bar-Or O (2010). Factors influencing the physical activity levels of youths with physical and sensory disabilities. Adapted Physical Activity Quarterly.

[ref-36] Martin Ginis KA, Ma JK, Latimer-Cheung AE, Rimmer J (2016). A systematic review of review articles addressing factors related to physical activity participation among children and adults with physical disabilities. Health Psychology Review.

[ref-37] Mendonca G, Cheng LA, Melo EN, de Farias Junior JC (2014). Physical activity and social support in adolescents: a systematic review. Health Education Research.

[ref-38] Moore LL, Lombardi DA, White MJ, Campbell JL, Oliveria SA, Ellison RC (1991). Influence of parents’ physical activity levels on activity levels of young children. The Journal of Pediatrics.

[ref-39] Ornelas IJ, Perreira KM, Ayala GX (2007). Parental influences on adolescent physical activity: a longitudinal study. International Journal of Behavioral Nutrition and Physical Activity.

[ref-40] Perkins K, Columna L, Lieberman L, Bailey J (2013). Parents’ perceptions of physical activity for their children with visual impairments. Journal of Visual Impairment & Blindness.

[ref-41] Peterson MS, Lawman HG, Wilson DK, Fairchild A, Van Horn ML (2013). The association of self-efficacy and parent social support on physical activity in male and female adolescents. Health Psychology.

[ref-42] Pirasteh A, Hidarnia A, Asghari A, Faghihzadeh S, Ghofranipour F (2008). Development and validation of psychosocial determinants measures of physical activity among Iranian adolescent girls. BMC Public Health.

[ref-43] Pluta B, Korcz A, Krzysztoszek J, Bronikowski M, Bronikowska M (2020). Associations between adolescents’ physical activity behavior and their perceptions of parental, peer and teacher support. Archives of Public Health.

[ref-44] Prieto LA, Meera B, Katz H, Hernandez MI, Haegele JA, Columna L (2024). Physical activity of children with visual impairments: intentions and behaviors of parents post intervention. Disability and Rehabilitation.

[ref-45] Prochaska JJ, Rodgers MW, Sallis JF (2002). Association of parent and peer support with adolescent physical activity. Research Quarterly for Exercise and Sport.

[ref-46] Qi J, Xu JW, Shao WD (2020). Physical activity of children with visual impairments during different segments of the school day. International Journal of Environmental Research and Public Health.

[ref-47] Robinson BL, Lieberman LJ (2007). Influence of a parent resource manual on physical activity levels of children with visual impairments. Re View.

[ref-48] Sit CHP, Huang WY, Yu JJ, Mckenzie TL (2019). Accelerometer-assessed physical activity and sedentary time at school for children with disabilities: seasonal variation. International Journal of Environmental Research and Public Health.

[ref-49] Sit CHP, Mckenzie TL, Cerin E, Chow BC, Yu J (2017). Physical activity and sedentary time among children with disabilities at school. Medicine & Science in Sports & Exercise.

[ref-50] Tatsuta W, Inayama T, Yamanaka E, Ohkawara K (2022). Assessment of physical activity and related factors among adults with visual impairments in Japan. International Journal of Environmental Research and Public Health.

[ref-51] Trost SG, Loprinzi PD, Moore R, Pfeiffer KA (2011). Comparison of accelerometer cut points for predicting activity intensity in youth. Medicine & Science in Sports & Exercise.

[ref-52] Trost SG, Sallis JF, Pate RR, Freedson PS, Taylor WC, Dowda M (2003). Evaluating a model of parental influence on youth physical activity. American Journal of Preventive Medicine.

[ref-53] van der Ploeg HP, van der Beek AJ, van der Woude LHV, van Mechelen W (2004). Physical activity for people with a disability: a conceptual model. Sports Medicine.

[ref-54] Voskuil VR, Robbins LB (2015). Youth physical activity self-efficacy: a concept analysis. Journal of Advanced Nursing.

[ref-55] Welk GJ (1999). The youth physical activity promotion model: a conceptual bridge between theory and practice. Quest.

[ref-61] World Health Organization (2020). WHO Guidelines on Physical Activity and Sedentary Behaviour.

[ref-56] Wu JT, Zhao XJ, Zhao WN (2022). The impact of physical exercise on negative emotions in college students: the mediating role of self-efficacy. Chinese Journal of Health Psychology.

[ref-57] Yao CA, Rhodes RE (2015). Parental correlates in child and adolescent physical activity: a meta-analysis. International Journal of Behavioral Nutrition and Physical Activity.

