# Supplementary material for: Associations between parental support and physical activity in children and adolescents with visual impairments: a mediating analysis of exercise self-efficacy
Source: PeerJ. 2026 Oct 28;14:e21871. doi: 10.7717/peerj.21871 (PMC13723195; doi:10.7717/peerj.21871)
Supplement: Supplementary file 1 [file peerj-14-21871-s005.pdf]

DOI:10.14036/j.cnki.cn11-4513.2016.04.020

首都体育学院学报
Journal of Capital University of Physical Education and Sports

第28卷 第4期/2016年7月
Vol.28 No.4 / July 2016
380~384

残疾青少年体力活动水平及其与运动自我效能的关系:基于加速度计的初步研究

Physical Activity Levels and Exercise Self-efficacy in Adolescents with Disability: A Preliminary Study Using Accelerometry

王　超[1],贺　刚[1],李建忠[2],郑　岩[1]
WANG Chao[1], HE Gang[1], LI Jianzhong[2], ZHENG Yan[1]

摘　要:以加速度计客观测量残疾青少年体力活动水平并分析其与运动自我效能的关系。方法:某特殊教育学校初一至高三的120名聋障及智障学生自愿参加本研究。研究对象体力活动水平以ActiGraph GT3X+加速度计测量,依据针对中国儿童、青少年确定的加速度计数界值计算其低强度体力活动(LPA)及中高强度体力活动(MVPA)时间。研究对象在教师的辅助下完成运动自我效能量表,并测试身高、体重等一般情况数据。以描述性统计分析研究对象平均每天LPA及MVPA时间及其在上学日、周末日的分布。运动自我效能与体力活动的关系采用控制年龄、性别、残疾类型3因素的偏相关分析进行统计。结果:58名提供了完整测试数据的研究对象(男生34人,女生24人;平均年龄(14.71±3.20)岁)被纳入数据分析。研究对象平均每天LPA及MVPA时间分别为(152.56±40.94) min/d及(30.28±13.19) min/d,仅有1.7%(男生2.9%,女生0%)达到世界卫生组织的体力活动推荐量(60 min/d MVPA),男生、女生周末日MVPA时间均低于上学日。偏相关分析显示,运动自我效能与研究对象周末日LPA及MVPA均呈正相关,而与上学日体力活动水平无关。结论:我国残疾青少年体力活动水平极低,尤以周末为著,提升残疾青少年运动自我效能可能有助于提高其周末日体力活动水平。

关键词:残疾;青少年;体力活动;运动自我效能;加速度计
中图分类号:G804.2　**文章编号**:1009-783X(2016)04-0380-05　**文献标志码**:A

Abstract: The purposes of this study were to objectively assess physical activity levels of adolescents with disability using accelerometry, and to investigate the association of physical activity and exercise self-efficacy. Methods: Participants of this study were 120 students with impaired hearing and intellectual disabilities recruited from grades 7~12 of a special education school. Physical activity levels of participants were measured by ActiGraph GT3X+ accelerometers. Time spent in light (LPA) and moderate-to-vigorous physical activity (MVPA) was calculated based on the cut-off points developed for Chinese youth. With the assistance of teachers, participants completed the exercise self-efficacy scale and body height and weight measurement. Descriptive statistics were conducted to analyze the daily LPA and MVPA time and its distribution on weekdays and weekend days. Partial correlation analysis were performed to examine the relationship between physical activity and exercise self-efficacy controlling for age, gender, and type of disability. Results: 58 participants (male students=34, female students=24, average age=14.71±3.20 years) who provided valid data were included in data analysis. Participants accrued 152.56±40.94 min/d of LPA and 30.28±13.19 min/d MVPA, respectively, with only 1.7% of whom (male 2.9%, female 0%) achieving the 60min/d MVPA recommended by WHO. Both male and female students do less MVPA on weekend days than on weekdays. Partial correlation analysis indicate that exercise self-efficacy is positively associated with LPA and MVPA on weekend days but not on weekdays. Conclusions: Physical activity levels of adolescents with disability in China are extremely low, especially on weekend days. Increasing exercise self-efficacy of adolescents with disability may facilitate the promotion of their physical activity levels on weekend days.

Keywords: disability; adolescent; physical activity; exercise self-efficacy; accelerometry

我国是儿童青少年残疾人口最多的国家。据2006年进行的第2次全国残疾人抽样调查数据,我国0~14岁的残疾人口达387万人,而6~14岁学龄残疾儿童青少年为246万人[1]。残疾为儿童青少年的身心健康带来严重影响的同时,也令其家庭背负着沉重的经济负担[2]。如何提高残疾儿童青少年的身心健康和生活质量成为摆在科研工作者面前的一项重要课题。

(C)1994-2023 China Academic Journal Electronic Publishing House. All rights reserved.　http://www.cnki.net

有研究显示，经常性地参与体力活动（包括体育锻炼及日常生活中的身体活动）有助于提高和维持残疾人群的身体功能和心理健康[3-4]；然而，调查显示，我国残疾儿童青少年体力活动水平低下，例如对河南省10个地市的1 200名3～14岁残疾儿童的调查显示，仅有19.4％的残疾儿童经常参与体育活动[5]。为提高残疾儿童青少年体力活动参与水平，发展有效的体力活动干预计划势在必行，其前提和关键是对其体力活动影响因素进行深入、准确的了解。

一项近期发表的系统综述显示，除身体及环境因素外，心理因素也是影响残疾儿童青少年参与体力活动的重要而稳定的因素[6]。随着残疾儿童步入青春期，其生理和心理都发生剧烈的变化，易产生自卑和羞耻感。研究表明，羞耻感是影响残疾学生参与体力活动的重要因素，而这一影响可能与其自我效能的变化有关[7]。追踪研究也证实，自我效能的改变与残疾人客观测量的体力活动水平变化相关，提示自我效能是影响残疾人体力活动参与的重要因素[8]；但在我国，自我效能与残疾儿童青少年体力活动的关系目前为止尚未见有研究报道。此外，截至目前，我国残疾儿童青少年体力活动领域的研究仍以问卷作为主要测量工具[5,7,9-10]，缺乏对其体力活动的客观、准确评价（例如，以加速度计进行客观测量），从而限制了体力活动影响因素研究的有效性。

因此，本研究的目的在于以加速度计客观测量残疾青少年体力活动水平，提供准确的客观数据，并初步分析运动自我效能对残疾青少年参与体力活动的影响。

1　研究对象与方法

1.1　研究对象

本研究以方便抽样的方法选取某特殊教育学校初一至高三的聋障及轻、中度智障学生作为研究对象。要求研究对象能够进行正常日常活动，身体发育健全、健康并无明显生理缺陷，基本能够正确理解测试要求，并在家长、老师的帮助下能够配合完成测试。事先向符合测试要求的学生家长派发邀请信，并详细介绍研究的目的意义、测试项目、测试流程、可能带来的危害及不便，最终共计120名学生获得家长同意参与本研究。

1.2　研究内容

1.2.1　人口统计学指标

包括出生日期、性别、残疾类型等，由测试学校负责提供。依据完成体力活动测试的时间计算年龄，对于测试完成当天已过生日者（含当天），年龄＝测试年－出生年；测试完成当天未过生日者，年龄＝测试年－出生年－1。

1.2.2　一般情况

包括身高、体重和身体体重指数。采用校正后的电子身高体重计测量身高、体重，身体体重指数（body mass index，BMI）以BMI ＝ 体重(kg)/身高的平方(m^2)进行计算。

1.2.3　体力活动水平

以美国产ActiGraph GT3X＋加速度计（ActiGraph，Ft. Walton Beach，USA）测量，这是目前国际上广泛应用于评价儿童、青少年体力活动的一种传感器。测试前将已完成数据初始化的加速度计发放给研究对象，由研究人员现场指导，用一个有弹性的带子戴在右髋部。研究对象被要求连续佩戴仪器7 d（包括5个上学日和2个周末日），除洗澡、游泳、睡觉外不能摘除。发放仪器后，研究人员联系测试学校老师和研究对象家长，对佩戴加速度计情况每天进行监督，以保证研究对象按时进行佩戴，尤其是周末时间。加速度计数据以ActiLife软件导出并进行有效性筛选：一个有效的佩戴日至少要包括8 h以上，每小时不少于40 min的非零数据；一个有效数据至少要包括2个有效上学佩戴日和1个有效周末佩戴日。依据针对中国儿童、青少年确定的加速度计数界值（＜2800 counts/min为LPA，≥2800 counts/min为MVPA）分别计算平均每天、上学日、周末日低强度体力活动（Light physical activity，LPA）及中等至高强度体力活动（Moderate-to-vigorous physical activity，MVPA）时间[11]。获得加速度计数据结果后，按照世界卫生组织（WHO）推荐的儿童、青少年应该进行不少于每天60 min MVPA的标准[12]，依据加速度计测量的MVPA时间，确定研究对象是否达到体力活动推荐量。

1.2.4　运动自我效能

以包含6个条目的5级Likert量表进行测量，该量表在青少年人群15日间隔重测信度为0.68（Pearson相关）[13]。研究对象在教师的辅助下回答他们在情绪低落时（条目一）、在每周的多数日子（条目二）、在家人和朋友要求做其他事时（条目三）、需要早起时（条目四）、有很多功课要做时（条目五）以及天气不好时（条目六）依然能够坚持进行运动的自信程度，答案选项包括由“非常不肯定”（1分）到“非常肯定”（5分）共5个等级。在获取每个条目评分基础上，依据6个条目的评分总和计算平均分值。

1.3　数据处理

以描述性统计报告研究对象平均每天体力活动时间及体力活动时间在上学日、周末日的分布。男、女性及智障、残障之间各指标差异以独立样本t检验比较；上学日与周末日间体力活动时间的差异以配对样本t检验比较。运动自我效能与体力活动的关系采用控制年龄、性别、残疾类型3因素的偏相关分析（partial correlation analysis）进行统计，按照Cohen标准，相关系数为±0.1、±0.3、±0.5分别对应弱、中等和强的效应量。将研究对象按照其运动自我效能所有条目的平均分值分为高、低2个水平（大于3为高水平，小于或等于3为低水平），以独立样本t检验比较高、低水平自我效能研究对象间的周末日体力活动水平差异。所有统计分析使用SPSS 20.0进行，显著性水平为$P<0.05$。

收稿日期：2016-06-07
基金项目：教育部人文社会科学研究青年基金项目（14YJC890025）。
第一作者简介：王超（1978-），女，河北秦皇岛人，博士，研究方向为青少年体力活动与健康。
作者单位：1.首都体育学院，北京 100191；2.秦皇岛市特殊教育学校，河北秦皇岛 066299
1. Capital University of Physical Education and Sports，Beijing 100191，China；2. Qinhuangdao Special Education School，Qinhuangdao 066299，China.

(C)1994-2023 China Academic Journal Electronic Publishing House. All rights reserved.　http://www.cnki.net

2 研究结果

2.1 研究对象基本情况

120名研究对象中有4人因仪器故障未获得体力活动测量数据,35人未能提供符合测试要求的完整有效体力活动数据。在81名提供了有效加速度计数据的研究对象中,58人(智障22人,聋障36人)完成运动自我效能问卷,被纳入最终数据分析。其中:男生34人,平均年龄(15.15±2.79)岁;女生24人,平均年龄(14.08±3.67)岁。具体数据见表1。

2.2 研究对象体力活动水平

研究对象中仅有1.7%(男生2.9%,女生0%)达到世界卫生组织的体力活动推荐量(60 min/d MVPA)。智障女生平均每天LPA与上学日LPA均显著低于聋障女生($P<0.05$),而智障男生周末日MVPA显著高于聋障男生($P<0.05$)。与上学日比较,聋障学生周末日LPA、MVPA均显著降低($P<0.05$),而智障学生上学日与周末日体力活动水平比较,未见显著性差异($P>0.05$)。智障女生平均每天MVPA及上学日MVPA均显著低于智障男生($P<0.05$),而聋障男、女生比较,未见显著性差异($P>0.05$),具体数据结果见表2。

表1 研究对象基本情况

参数	总样本(n=58)	男生(n=34)	女生(n=24)
年龄/岁	14.71±3.20	15.15±2.79	14.08±3.67
身高/cm	163.97±10.80	168.68±10.13	157.29±7.92
体重/kg	54.67±12.65	58.59±12.98	49.13±10.01
BMI/(kg/m^2)	20.14±3.46	20.48±3.83	19.66±2.88
智障人数(%)	22(37.9)	15(44.1)	7(29.2)
聋障人数(%)	36(62.1)	19(55.9)	17(70.8)

表2 研究对象体力活动水平一览表

参数		LPA/(min/d)			MVPA/(min/d)			满足WHO推荐量情况/%		
		平均每天	上学日	周末日	平均每天	上学日	周末日	平均每天	上学日	周末日
总样本	总体	152.56±40.94	154.68±43.11	145.06±49.40	30.28±13.19	32.35±14.84	25.10±17.55△	1.7	1.7	6.9
	智障	147.07±38.31	144.98±42.16	152.03±52.84	32.73±18.26	33.47±20.32	31.04±23.48	4.5	4.5	13.6
	聋障	155.92±42.65	160.61±43.18	140.80±47.43△	28.79±8.79	31.66±10.48	21.46±11.62△	0	0	0
男生	总体	153.88±43.76	156.14±46.76	145.85±49.12	34.38±13.76	36.77±15.97	28.61±19.47△	2.9	2.9	11.8
	智障	156.23±42.72	155.73±46.67	157.23±53.96	38.85±18.35	39.80±21.37	37.33±24.08	6.7	6.7	20
	聋障	152.02±45.64	156.47±48.11	136.86±44.33△	30.85±7.46	34.37±9.95	21.72±11.42*△	0	0	5.3
女生	总体	150.70±37.42	152.60±38.22	143.95±50.84	24.48±9.98#	26.08±10.50#	20.13±13.26△	0	0	0
	智障	127.46±14.84	121.93±15.21	140.88±52.60	19.61±9.35#	19.92±8.05#	17.58±16.36	0	0	0
	聋障	160.27±39.96*	165.23±37.84*	145.21±51.69△	26.48±9.78	28.62±10.52	21.17±12.18△	0	0	0

注:*为与智障比较,差异具有显著性($P<0.05$);△为与上学日比较,差异具有显著性($P<0.05$);#为与男生比较,差异具有显著性($P<0.05$)。下同。

2.3 研究对象运动自我效能

运动自我效能每个条目评分介于1~5分,条目一至条目六的所有条目平均分值是(2.50±0.88)。男生与女生的各个条目及所有条目平均分比较,未见显著性差异($P>0.05$)。智障男生条目三、条目四、条目五的评分及平均分值均显著低于聋障男生($P<0.05$),智障女生在条目一、条目二、条目四、条目五的评分及平均分值均显著低于聋障女生($P<0.05$),具体数据结果见表3。

表3 研究对象的运动自我效能

参数	总样本			男生			女生		
	总体	智障	聋障	总体	智障	聋障	总体	智障	聋障
条目一	2.43±1.04	2.09±1.23	2.64±0.87	2.50±1.16	2.33±1.35	2.63±1.01	2.33±0.87	1.57±0.79	2.65±0.70*
条目二	2.76±1.17	2.32±1.32	3.03±1.00*	2.79±1.12	2.60±1.45	2.95±0.78	2.71±1.27	1.71±0.76	3.12±1.22*
条目三	2.41±1.19	1.82±1.05	2.78±1.12*	2.50±1.13	1.93±1.16	2.95±0.91*	2.29±1.27	1.57±0.79	2.59±1.33
条目四	2.72±1.50	1.73±1.20	3.33±1.33*	2.88±1.53	1.80±1.26	3.74±1.15*	2.50±1.44	1.57±1.13	2.88±1.41*
条目五	2.53±1.22	1.95±1.13	2.89±1.14*	2.59±1.10	2.13±1.13	2.95±0.97*	2.46±1.38	1.57±1.13	2.82±1.33*
条目六	2.14±1.19	1.91±1.19	2.28±1.19	2.26±1.24	2.00±1.25	2.47±1.22	1.96±1.12	1.71±1.11	2.06±1.14
平均分值	2.50±0.88	1.97±0.98	2.82±0.62*	2.59±0.84	2.13±1.04	2.95±0.37*	2.38±0.93	1.62±0.82	2.69±0.80*

2.4 研究对象体力活动水平与运动自我效能的偏相关分析

控制年龄、性别、残疾类型3因素的偏相关分析显示,运动自我效能中条目四(r=0.37)、条目五(r=0.37)评分及平均分值(r=0.35)与周末日LPA均具有中等相关性($P<0.05$),条目二(r=0.30)、条目三(r=0.31)评分及平均分值(r=0.39)与周末日MVPA具有中等相关性($P<0.05$),具体数据结果见表4。

2.5 不同运动自我效能的周末日体力活动水平比较

因偏相关分析显示运动自我效能仅与周末日体力活动水平相关,在此仅比较高、低运动自我效能水平研究对象间周末日体力活动水平差异。结果显示高运动自我效能研究对象的周末日LPA显著高于低运动自我效能研究对象($P<0.05$),MVPA数值上有同样的变化趋势;但统计学上未见显著性差异($P>0.05$),具体数据结果

(C)1994-2023 China Academic Journal Electronic Publishing House. All rights reserved. http://www.cnki.net

见表 5。

表 4　研究对象体力活动水平与运动自我效能的偏相关分析

参数	平均每天				上学日				周末日			
	LPA		MVPA		LPA		MVPA		LPA		MVPA	
	r	*P*	*r*	*P*	*r*	*P*	*r*	*P*	*r*	*P*	*r*	*P*
条目一	0.04	0.77	−0.07	0.60	0.05	0.74	−0.13	0.33	0.04	0.76	0.12	0.38
条目二	0.23	0.09	0.03	0.80	0.19	0.17	−0.10	0.47	0.25	0.07	0.30	0.02
条目三	−0.07	0.63	0.12	0.40	−0.08	0.54	0.01	0.92	0.03	0.81	0.31	0.02
条目四	0.22	0.11	0.05	0.70	0.15	0.29	−0.07	0.59	0.37	0.01	0.29	0.03
条目五	0.23	0.08	−0.03	0.84	0.16	0.25	−0.16	0.25	0.37	0.00	0.29	0.03
条目六	0.23	0.09	0.02	0.86	0.18	0.19	−0.09	0.52	0.28	0.04	0.24	0.08
平均分值	0.23	0.09	0.03	0.81	0.16	0.23	−0.13	0.33	0.35	0.01	0.39	0.03

表 5　不同运动自我效能的周末日体力活动水平比较

运动自我效能水平	周末日	
	LPA/(min/d)	MVPA/(min/d)
低水平(n=50)	139.31±46.10	24.49±16.39
高水平(n=8)	180.97±57.32&	28.91±24.65

注：& 为与低水平运动自我效能比较，差异具有显著性(P<0.05)。

3　讨论

有研究显示，采用客观测量方法获得残疾人群体力活动数据，结果较为准确和一致[14]。本研究采用加速度计这一国际通用的体力活动测量工具，首次提供了我国残疾青少年体力活动水平的客观数据，尽管样本量较小，但较之前采用问卷调查方法更加科学准确，具有较强的参考价值。本研究发现残疾学生体力活动水平严重不足，总体 MVPA 时间仅有约 30 min/d，仅有 1.7%的学生能够达到 WHO 的体力活动推荐量。这一比例低于采用同一款加速度计测得的我国正常发育青少年(5.5%～12%满足 WHO 体力活动推荐量)[15]，该结果与国外针对智障儿童青少年开展的体力活动研究结果基本一致[14]；另一方面，该比例也低于我国采用问卷调查得到的残疾儿童青少年经常参与体育活动的比例(19.4%)[5]，提示既往问卷调查可能高估了我国残疾儿童青少年体力活动水平。从体力活动时间来看，本研究测得的残疾学生体力活动时间远低于国外残疾儿童。例如：Ryan 等[16]采用加速度计测得脑瘫儿童 MVPA 时间约为 50 min/d；Pitetti 等[17]以心率计测得智障儿童于学校期间的 MVPA 时间高达 83.5 min/d。尽管与国外研究的测试方法并不完全一致，可能导致比较研究存在一些偏差，但是考虑到与我国正常发育青少年体力活动测试方法完全一致，完全具有可比性，足以证实我国残疾青少年体力活动缺乏的事实；因此，急需有效的干预方案和措施以切实提高残疾儿童青少年的体力活动水平。

运动自我效能反映个体对于克服各种困难继续坚持规律运动的自信心。残疾青少年由于身体的缺陷，在参与体力活动过程中易出现不自信乃至自卑心理。已有研究显示，身体障碍儿童参与活动(包括体力活动)的意愿虽然受限于运动能力，但更主要的阻碍因素是其较低的自我效能水平[18]。本研究的结果亦证实，残疾学生的运动自我效能得分较低(2.5 分)，并且智障学生运动自我效能明显低于聋障学生，提示智障青少年智力水平低下的同时可能伴有更严重的自信心缺乏及成功预期低下。

此外，本研究结果显示运动自我效能与残疾青少年周末日 LPA 及 MVPA 均呈正相关，而与上学日体力活动水平无关，并且进一步的数据统计分析显示运动自我效能水平高的残疾学生其周末日 LPA 确实显著高于运动自我效能低的残疾学生，证实运动自我效能对残疾青少年参与体力活动具有积极影响。自我效能与体力活动的关系在残疾成人的研究中已经得到了证实，Motl 等[8]的追踪研究表明残疾人自我报告及客观测量的体力活动水平变化均与其自我效能的改变呈正相关；此外，另有研究证实自我效能也在其他因素(如社会支持)对体力活动的影响中发挥重要的中介作用[19]。来自残疾儿童青少年的研究结果极为有限，Cairney 等[20]以问卷法调查了 590 名发育协调障碍的儿童，发现儿童的一般自我效能与其体力活动水平有明确的相关性。本研究还发现运动自我效能与残疾青少年周末日及上学日体力活动的相关性存在差异，推测与残疾青少年在上述时间体力活动参与模式有关。具体来说，上学日残疾学生主要在学校进行有组织的体力活动(如体育课、课外活动等)，属于强制参与性体力活动，不同自我效能水平学生间体力活动不易表现出差异；而在周末日，残疾学生体力活动形式为自主性的自由活动，自我效能水平高者可能更多地参与各种强度的体力活动。在体力活动满足 WHO 推荐量情况方面，间接证实了这一推测。尽管周末日整体 LPA、MVPA 水平降低，但是满足 WHO 体力活动推荐量的百分比竟然提高。这一反差说明有少数智障青少年(主要是男生)在可以自主安排活动的周末日具有比上学日更高的体力活动水平。

本研究在我国较早地以加速度计对残疾青少年体力活动水平进行客观描述，并初步证实运动自我效能与残疾青少年体力活动水平的关系，但本研究尚有以下局限性：首先，由于研究对象的特殊性，本研究采用方便抽样且样本量有限，在有限的样本量范围内因为残疾学生配合度差，导致样本有效性较低(不足 50%有效)；其次，本研究纳入的残疾青少年体力活动影响因素指标仅有运动自我效能一项，其他心理、社会乃至环境因素对残疾青少年体力活动的影响有待今后的研究进一步阐明。

(C)1994-2023 China Academic Journal Electronic Publishing House. All rights reserved.　http://www.cnki.net

4 结论

本研究发现我国特殊教育学校残疾青少年体力活动水平极低，急需发展有效的干预手段促进其体力活动参与水平。运动自我效能与残疾青少年周末日体力活动水平密切相关，针对周末日的体力活动干预方案在设计时可考虑纳入提升运动自我效能的成分。鉴于本研究的局限性，建议今后的研究采用随机或整群抽样的大样本量研究设计，并综合探究心理、社会及环境等因素对残疾青少年体力活动的影响。

参考文献：

[1] 国家统计局，第二次全国残疾人抽样调查领导小组. 第二次全国残疾人抽样调查主要数据公报[EB/OL]. [2016-06-15]. http://www.stats.gov.cn/tjsj/ndsj/shehui/2006/html/fu3.htm.

[2] 熊妮娜，杨丽，于洋，等. 孤独症、肢体残疾、智力残疾儿童家庭经济负担调查[J]. 中国康复理论与实践，2010(8)：788.

[3] MOBILY K E. Role of exercise and physical activity in therapeutic recreation services[J]. Therapeutic Recreation Journal，2009，43(2)：9.

[4] SRINIVASAN S M，PESCATELLO L S，BHAT A N. Current perspectives on physical activity and exercise recommendations for children and adolescents with autism spectrum disorders[J]. Physical Therapy，2014，96(6)：1.

[5] 郭照德. 河南省残疾儿童参与体育活动现状调查及对策[J]. 实用儿科临床杂志，2010(13)：1030.

[6] RU L，CINDY H S，JANE J Y，et al. Correlates of physical activity in children and adolescents with physical disabilities：A systematic review[J]. Preventive Medicine，2016(89)：184.

[7] 亓圣华. 残疾学生羞耻感与体育锻炼的关系研究[J]. 北京体育大学学报，2014，37(1)：92.

[8] MOTL R W，MCAULEY E，SANDROFF B M. Longitudinal Change in Physical Activity and Its Correlates in Relapsing-Remitting Multiple Sclerosis[J]. Physical Therapy，2013，93(8)：1037.

[9] 左燕. 湘西特殊教育学校学生课外体育锻炼的现状分析[J]. 内江科技，2010(3)：148.

[10] 司琦，陈红玉，刘海群，等. 促进弱势群体参与体育锻炼的干预研究：以听力残疾学生为例[J]. 体育科学，2010，30(7)：32.

[11] ZHU Z，CHEN P，ZHUANG J. Intensity classification accuracy of accelerometer-measured physical activities in Chinese children and youth[J]. Research Quarterly for Exercise and Sport，2013，84(sup2)：4.

[12] WHO. Global Recommendations on Physical Activity for Health[M]. Press：Geneva，2010：25.

[13] PIRASTEH A，HIDARNIA A，ASGHARI A，et al. Development and validation of psychosocial determinants measures of physical activity among Iranian adolescent girls[J]. BMC Public Health，2008(8)：150.

[14] HINCKSON E，CURTIS A. Measuring physical activity in children and youth living with intellectual disabilities：a systematic review[J]. Research in Developmental Disabilities，2013，34(1)：72.

[15] CHAO W，PEIJIE C，JIE Z. A national survey of physical activity and sedentary behavior of Chinese city children and youth using accelerometers[J]. Research Quarterly for Exercise and Sport，2013，84(sup2)：25.

[16] RYAN J M，FORDE C，HUSSEY J M，et al. Comparison of patterns of physical activity and sedentary behavior between children with cerebral palsy and children with typical development[J]. Physical Therapy，2015，95(12)：1612.

[17] PITETTI K，BEETS M，COMBS C. Physical activity levels of children with intellectual disabilities during school[J]. Medicine and Science in Sports and Exercise，2009，41(8)：1580.

[18] ENGEL-YEGER B，KASIS A H. The relationship between Developmental Co-ordination Disorders，child′s perceived self-efficacy and preference to participate in daily activities[J]. Child：Care，Health and Development，2010，36(5)：670.

[19] PETERSON J J，LOWE J B ，PETERSON N A，et al. Paths to leisure physical activity among adults with intellectual disabilities：Self-Efficacy and social support[J]. American Journal of Health Promotion，2008，23(1)：35.

[20] CAIRNEY J，HAY J A，FAUGHT B E，et al. Developmental coordination disorder，generalized self-efficacy toward physical activity，and participation in organized and free play activities[J]. Journal of Pediatrics，2005，147(4)：515.

(C)1994-2023 China Academic Journal Electronic Publishing House. All rights reserved. http://www.cnki.net
