# Supplementary material for: Associations between parental support and physical activity in children and adolescents with visual impairments: a mediating analysis of exercise self-efficacy
Source: PeerJ. 2026 Oct 28;14:e21871. doi: 10.7717/peerj.21871 (PMC13723195; doi:10.7717/peerj.21871)
Supplement: Supplementary file 2 [file peerj-14-21871-s008.pdf]

BMC Public Health

Research article

Open Access

Development and validation of psychosocial determinants measures of physical activity among Iranian adolescent girls

Ashraf Pirasteh†1, Alireza Hidarnia*†1, Ali Asghari†3, Soghrate Faghihzadeh†2 and Fazlollah Ghofranipour†1

Address: 1Department of Health Education, Tarbiat Modares University, Tehran, Iran, 2Department of Vital Statistic, Tarbiat Modares University, Tehran, Iran and 3Department of Psychology, Shahed University, Tehran, Iran

Email: Ashraf Pirasteh - pirasteh2002@yahoo.com; Alireza Hidarnia* - hidarnia@modares.ac.ir; Ali Asghari - asghari_a@yahoo.com; Soghrate Faghihzadeh - faghihz@modares.ac.ir; Fazlollah Ghofranipour - ghofranf@modares.ac.ir

* Corresponding author †Equal contributors

Published: 7 May 2008

BMC Public Health 2008, **8**:150 doi:10.1186/1471-2458-8-150

Received: 6 September 2007
Accepted: 7 May 2008

This article is available from: http://www.biomedcentral.com/1471-2458/8/150

© 2008 Pirasteh et al; licensee BioMed Central Ltd.
This is an Open Access article distributed under the terms of the Creative Commons Attribution License (http://creativecommons.org/licenses/by/2.0), which permits unrestricted use, distribution, and reproduction in any medium, provided the original work is properly cited.

Abstract

Background: The present study aimed at assessing the psychometric properties of psychosocial determinants of physical activity-related measures in Iranian adolescent girls.

Methods: Several measures of psychosocial determinants of physical activity were translated from English into Persian using the back-translation technique. These translated measures were administered to 512 ninth and tenth-grade Iranian high school students.

Results: The results of a series of factor analysis showed that the self-efficacy scale contained a single factor, the social support scale contained two factors: family support and friend support, the physical activity 'pros & cons' scale contained two factors: physical activity pros scale and physical activity cons scale, the change strategies scale contained a single factor, the environment scale also contained a single factor. Chronbach's alphas, mean inter-item correlations and test-retest coefficients showed that these solutions were reliable.

Conclusions: These preliminary results provide support for using the mentioned scales to measure psychosocial determinants of physical activity in Iranian adolescent girls.

Background

Promotion of physical activity level is one of the most important and effective strategies for reducing the risk of several chronic diseases including cardiovascular diseases, non-insulin-dependent diabetes mellitus, osteoporosis, obesity and some types of cancer [1].

Physical activity habits fostered and developed during the early stages of life may be expected to persist into adulthood, reducing the incidence of chronic diseases associated with a sedentary lifestyle in later life [2]. Given the age-related decline of physical activity, adolescence seems to be a critical period [3].

Promotion of physical activity level among adolescents can be desired by behavioral interventions. More effective interventions are needed because half of individuals who initiate a physical activity program drop out within six months [4].

Data from three national surveys among Iranian adults have shown that more than 80% of the Iranian popula-

tion is physically inactive [5]. A few local studies performed in Iranian young people have revealed a similar pattern. The decrease in physical activity levels is suggested to be as a result of increases in time spent watching television and playing computer games, as well as of a decrease in opportunities for physical activity in schools and communities [6].

A major issue in physical activity programs and research among adolescents is the accurate measurement psychosocial determinants of physical activity which may contribute to physical activity in this population. This has led healthcare professionals and researchers to develop exercise interventions based on theoretical models of behavior change in an attempt to increase physical activity levels [7].

To understand the levels of physical activity among individuals, various researchers have identified a number of promising variables that may influence levels of physical activity. These variables include demographics, cognition, behaviour, social environment, and physical environment. In intervention programmes, cognitive variables are particularly targeted, because they may be more amenable to change than the less mutable variables such as age and income [8]. Although researchers have claimed that the cognitive variables are responsible for a considerable proportion of variance in physical activity levels, the measurement of these variables is not frequently standardized.

In this study, the instruments were used from PACE-Adolescent Physical Activity Survey and translated from English into Persian using the back-translation technique. Social Cognitive Theory (SCT) and the Transtheorical Model (TTM) guided instrument development [9,10].SCT is relevant for designing health education and health behavior programs and explains how people acquire and maintain certain behavioral patterns. The theory can also be used for providing the basis for intervention strategies [11].

The Transtheoretical Model (TTM) of behavior change can also provide a useful framework for examining the issue of adoption and maintenance of physical activity with adolescents [12]. The TTM is an effective way of depicting individual's readiness to engage in a variety of healthy behaviors including smoking and alcohol cessation, diet change and, more recently, engaging in an exercise or in a physical activity program [13].

There are no theoretically based instruments in the literature that measure physical activity related psychosocial determinates among Iranian adolescents. Thus, the present study is the first research for the development of physical activity related psychosocial determinant measures.

It also examines the psychometric characteristics of several physical activity-related psychosocial determinant measures in Iranian adolescent girls. Acknowledging the low physical activity during adolescence, standardized, reliable and valid measures of influence of physical activity for this population is essential. In this study, some 512 high school students were administered the questionnaires of physical activity along with other measures to evaluate their reliability and validity for this population. We conducted tests of internal consistency, test-retest reliability and factor analysis in constructs of physical activity self-efficacy, physical activity social support, physical activity pros and cons, physical activity change strategies, and physical activity environmental factors. Consistent with the initial test development, we predicted a good internal consistency among the scales, and high test-retest reliability.

Methods

Participants

Participants were female students who meet the inclusion criteria of the study (i.e., studying in high school (9th or 10th grades) and being able to attend two survey sessions). The eligible subjects were recruited from 12 high schools in Tehran. The age of participants ranged from 15 to 17 years with average age 16.15 years (SD = 0.77). A total of 545 students were recruited into the study, 33 subjects were omitted from the analysis due to missing data on one or more of the determinants physical activity items of interest. Popular textbooks on factor analysis give specific advice on sample size for factor analysis, the required variable to subject ratio lies between 1:5 and 1:10 [14]. The present paper reports the results of the validation process of a Persian version of a series of scales measuring psychosocial determinants of physical activity in a group of Iranian adolescents in Tehran. The most important research question was: "*Are the questionnaires a valid and reliable measure for Iranian adolescents?*"

Ethical consideration

Permission to use the original scales was obtained from the author. The approval for the use of human subjects was obtained from the Iranian Ministry of Education. The ethical committee of Tarbiat Modares University approved the study. The participants were told about the general nature of the study and were assured of the confidentiality of the data and informed consent for the study was obtained from the entire subject.

Instrument Development

Samuel Messick *(1995)* believes in six aspects of construct validation including content, substantive, structural, gen-

eralizability, external, and consequential as they apply to performance assessment. Also, Samuel Messick argues that 'it is not sufficient merely to select tasks that are relevant to the construct domain. In addition, the assessment should assemble tasks that are representative of the domain... The intent is to insure that all important parts of the construct domain are covered [15].'

In the research literature of nursing and other health care professions, factor analysis is most often used as a part of the instrument development process. Factor analysis may be a vital step in creating a new measurement tool; it is a method for organizing the items into factors. A factor is a group of items that could be said to be related to each other [16].

After comprehensive literature review on the existing instruments of measuring physical activity determinants in adolescents, we used measures of physical activity related psychosocial determinants that have previously been adapted and developed among the American adolescents by Norman & Sallis [17]. First, translation and back translation procedure based on Brislin's model [18] was used to develop culturally equivalent questionnaires. Two bilingual experienced health educators translated the questionnaires into Persian and another two bilingual health educators back translated them independently. The researchers and the four translators discussed the clarity of the translation work and examined discrepancies between the two versions, and finally amended a few items to ensure the appropriateness of the translation [19]. For example, "Dedicate a specific time for doing exercise or physical activity on most days of the week?" instead of "Set aside time for physical activity on most days of the week." translated in the physical activity self-efficacy scale. The final versions of the translated questionnaires are presented in Additional file 1.

A panel of eight Iranian experts in the areas of health education and clinical psychology were asked to quantify the clarity linguistic appropriateness of the translated questionnaires (content validity). The panel members were asked to evaluate the pilot instrument for the appropriateness and relevance of the items. Furthermore, the expert panel was asked to evaluate item wording, response format, and instrument length.

A pilot study was conducted to test whether the physical activity questionnaires were easy to read and to comprehend by the students. A convenience sample of 12- students completed the physical activity questionnaires and gave comments on their understanding of the items. The changes made to the original version include adoption of age-appropriate words and the development of a format more appealing to adolescence girls.

Measures

Self-efficacy

This variable asked the individuals about their confidence in being able to carry out a regular schedule of exercise as well as the barriers they perceived in exercising. A six-item physical activity self-efficacy scale was used based on the previous scales [20,21].The participants responded to each item on a 5-point Likert scale ranging from one "*I'm sure I can't*" to five "*I'm sure I can*".

Decisional balance

Decisional balance consisted of two constructs labeled the 'Pros' and 'Cons' of change that address cognitive and motivational aspects of human decision-making. Marcus, Rakowski and Rossi [22] modified Velicer's decisional balance inventory for smoking cessation to apply to exercise behavior and demonstrated good internal consistency and concurrent validity with stage of change for exercise. In this study a 10-item physical activity pros and cons scale (5 pros and 5 cons) was used and the participants responded to each item on a 5-point Likert scale ranging from one "*not important*" to five "*extremely important*".

Family support

Four items of family support on physical activity assessed family influences on physical activity [23]. The items asked the frequency a household member encouraged, participated, provided transportation, and watched physical activity. The items were:

(1) Watched you participate in physical activity or play sports? (2) Encouraged you to do sports or physical activity? (3) Provided transportation to a place where you can do physical activity or play sports? (4) Done a physical activity or played sports with you?

Items were asked in reference to a typical week and participants responded using a 5-point scale from one "*Never*" to five "*Every Day*".

Friend support

Items similar to the family support items assessed friend support related to physical activity. The five items assessed the frequency that friends provided encouragement and support for participating in physical activity. The items were: (1) Do your friends encourage you to do sports or physical activities?, (2) Do your friends do physical activity or play sports with you?, (3) Do your friends or classmates tease you about not being good at physical activities or sports?, (4) Do your friends ask you to walk or bike to school or to a friend's house? And (5) Do your friends tell you that you are doing well in physical activities or sports? The items were asked in reference to a typical week and the participants responded using a 5-point scale from one "*Never*" to five "*Every Day*".

Change strategies

The change strategies were similar to the constructs described as processes of change in the Transtheoretical Model [24] and were based on the items developed by Saelens, Gehrman, Sallis, Calfas, Sarkin and Caparosa [25]. Some fifteen items were used that reflect thoughts, feelings, and activities people may use when making a behavior change. The response format assessed how often each strategy was used by a 5-point Likert scale ranging from one "*Never*" to five "*Many Times*".

Environment

The measure of perceived environment that assessed the neighborhood environment in terms of facilitating physical activity included four items rated on a 5-point scale with anchors of one "*Disagree a lot*" and five "*Agree a lot*". The items were: (1) There are enough supplies and pieces of sports equipment (like balls, bicycles, skates) At home to use for physical activity; (2) There are playgrounds, parks or gyms close to my home or that I can get to easily; (3) It is safe to walk or jog alone in my neighborhood during the day; and (4) It is difficult to walk or jog in my neighborhood because of things like traffic, no sidewalks, dogs and gangs. The item number four was reverse-scored before all analyses.

These items were originally from the Amherst Health and Activity Study [23].

Data analysis

Each scale's reliability was estimated by calculating its internal consistency and test-retest stability. Internal consistency measured by coefficient alpha is the proportion of a scale's total variance that is attributable to a common source, the true score of a latent variable underlying the items [26]. A minimal reliability of 0.70 was considered sufficient to consider the scale useful and worth efforts at further refinement to reduce the scale's measurement error [27].

Another estimate of a scale's reliability is its temporal stability assessed by a test-retest design. The following standards were used to evaluate the reliability coefficients: (1) less than 0.00, poor; (2) 0.00–0.20, slight; (3) 0.21–0.40, fair; (4) 0.41–0.60, moderate; (5) 0.61–0.80, substantial; and (6) 0.81–1.00, excellent [28].

In this study, exploratory factor analysis (EFA) was used to summarize the data by grouping the intercorrelated variables together. Most often, this occurs in the early stages of research. The direct purpose of exploratory factor analysis (EFA) is to reduce a set of data so that it may be described and used easily. Other purposes include instrument development and theory construction [16].Principal components analysis with oblique or varimax rotation was conducted on each scale using data [29].

Results

Demographic characteristics of the participating girls are shown in Table 1. Average age of the girls was 15.74 years (SD = 0.77) and the average BMI was 20.91 kg/m^2. Household income was unfairly distributed across the four income categories. Some %62.8 of the participating girls' family had < $320 household income, %48.5 (n = 248) of fathers had completed high school, with %16.8 (n = 86) completing a college or graduate degree, and %54 (n = 276) of mothers had completed high school, with %09.0 (n = 46) completing a college or graduate degree.

Analysis approach

The sample size of 512 was sufficient to produce reliable correlation coefficients so that popular textbooks on factor analysis give specific advice on sample size for factor analysis. The required variable to subject ratio lies between 1:5 and 1:10 (14, 30).

Prior to performing Principal Components Analysis (PCA), the suitability of data for factor analysis was assessed. An inspection of the correlation matrix in each subscale revealed that most of the correlations were greater than 0.30, therefore, some clustering of items was expected and exploratory factor analysis was deemed appropriate in the early stage of research [31]. The Kaiser-Meyer- Olkin Measures of Sampling Adequacy value for examined scales ranged from 0.61 to 0.93, exceeding the recommended value of 0.60 [31] and the Bartlett's test of

Table 1: Characteristics of the participating girls

	Mean (SD)
Age (years)	15.74 (0.77)
Weight	54.22 (11.39)
Height	161.51 (10.88)
BMI	20.91
Father education (%)	
Some high school	31.5
High school graduate	48.5
College or graduate degree	16.8
Up BM	3.2
Mother education (%)	
Some high school	35.6
High school graduate	54.0
College or graduate degree	09.0
Up BM	1.4
Household income (%)	
< $320	62.8
$321–$550	25.8
$551–1100	9.6
> $1100	1.8

sphericity [32] reached statistical significance (P < 0.001), supporting the factorability of the correlation (Table 2). Thus, Principal Component Analysis (PCA) was used to identify scales' dimensions in this study. The decision between orthogonal and oblique rotation was made, examining the correlations among the factors [31]. The results of factor analysis are presented here:

For physical activity self-efficacy (Table 3), one factor was identified which was accounted for 55% of the variability in the items. The internal consistency estimate (alpha = 0. 84) was excellent and the test-retest reliability coefficient (r = 0.68) was substantial.

Two sub-scales were identified for the physical activity social support (Table 4), family support and friend support. These two factors accounted for 55% of the variability in the items. The internal consistency estimate for the family support scale was substantial (alpha = 0.72), as was the internal consistency estimate for the friend support scale (alpha = 0.77). The test-retest reliability of both scales was moderate (r = 0.56 and r = 0.54, respectively).

For the physical activity decisional balance (Table 5), two sub-scales of pros and cons were also identified, accounting for 50% of the variability in the items. The internal consistency estimate for the pros scale was substantial (alpha = 0.81), as was the internal consistency estimate for the cons scale (alpha = 0.69). The test-retest reliability of both scales was moderate (r = 0.44 and r = 0.36, respectively).

Principal Component Analysis (PCA) with oblique was performed on the students' responses to the 15 change strategies items. Oblique rotation, which allows the factors to be statistically related [31], was used because it was expected that the factors underlying change strategies would be correlated in reality. An initial analysis with principal component analysis was conducted to identify the number of factors with eigenvalues of 1.0 or greater, which is an estimate of the maximum number of stable factors [31]. The scree test [33], suggested the existence of factors.

Table 2: KMO* & Bartlett's test of sphericity psychosocial determinants of physical activity

	KMO	Bartlett's test (significance)
Self-efficacy	0.88	1043.051 (*p* = 0.00001)
Social support	0.79	1181.322 (*p* = 0.00001)
Pros & Cons	0.79	1153.842 (*p* = 0.00001)
Change strategies	0.93	2243.043 (*p* = 0.00001)
Environment	0.61	0387.790 (*p* = 0.00001)

*Kaiser-Meyer- Olkin

Table 3: Factor analysis for physical activity self-efficacy scale (N = 512)

Factor 1: Physical Activity Self-efficacy	Factor loadings
Be physical activity even it raining or hot	0.79
Get up early, even on weekends, to do physical activity	0.79
Set aside time for physical activity on most days	0.77
Be physical activity even I have a lot of schoolwork	0.75
Be physical activity even my family want me to do something else	0.71
Be physical activity even I feel sad or stress	0.65
Eigen value	3.35
% variance explained	55.97
Choronbach's alpha	0.84
Mean inter item correlation	0.47
Pearson test-retest*	0.68

*Test-retest stability with a 15-day interval (n = 93)

The eigenvalues for the first 2 consecutive components were 5.70 and 1.06. Examination of the eigenvalues greater than 1 indicated that a 2-factor solution may be appropriate. The examination of the scree plot also suggested that 2 dimensions underlie change strategies scale. Although these two methods are the most popular heuristic, they are potentially unreliable [[34,31], and [35]]. For example, Zwick and Velicer have argued that using eigenvalues greater than 1 to determine the number of factors to extract leads to 'overfactoring', it remains more factors

Table 4: Factor analysis for physical activity social support scale (N = 512)

	Factor1(Factor loadings)	Factor2(Factor loadings)
Friend support		
Tease from your friends	0.80	
Ask from your friends to walk	0.77	
Tell you that you are doing well	0.73	
Do physical activity with you	0.70	
Encourage you to do physical activity	0.65	
Family support		
Done with you		0.73
Provided transportation		0.73
Encourage you to do physical activity		0.72
Watched you		0.72
Eigen value	2.62	2.32
% variance explained	29.18	25.83
Choronbach's alpha	0.77	0.72
Mean inter item correlation	0.41	0.40
Pearson test-retest*	0.56	0.54
	Factor1(Factor loadings)	Factor2(Factor loadings)

* Test-retest stability with a 15-day interval (n = 93)

Table 5: Factor analysis for physical activity Pros & Cons scale (N = 512)

	Factor1(Factor loadings)	Factor2(Factor loadings)
Pros scale		
More energy	0.79	
Feel better	0.76	
Help to stay fit	0.72	
Parents would be happy	0.72	
Have fun with my friends	0.70	
Cons scale		
Takes time from being with my friends		0.70
Too much help from my parents		0.65
There is too much to learn		0.64
Don't like physical activity to make me feel		0.63
Feel embarrassed		0.42
Eigen value	3.07	1.98
% variance explained	30.70	19.88
Choronbach's alpha	0.81	0.69
Mean inter item correlation	0.46	0.23
Pearson test-retest*	0.44	0.36

* Test-retest stability with a 15-day interval (n = 93)

than is optimally required. In this study parallel analysis (PA) [36] was employed to ascertain the optimal number of factors to extract. The PA requires the researcher to randomly generate a raw data matrix on the same 'rank' as the actual raw data matrix. For example, if one had a 1-to-5 Likert scale data for 512 subjects on 15 variables, a 512-by-15 raw data matrix consisting of 1s, 2s, 3s, 4s and 5s would be generated. These random data can be factor analysed to produce a set of eigenvalues. The eigenvalues associated with the matrix of association based on observed data are also computed. The number of extractable factors is equal to the number with observed eigenvalues greater than the point on the plot where the observed and random eigenvalues cross [[34,36], and [37]].

Using the procedure recommended by Thompson and Daniel [37], 50 random data sets were generated of the same order of change strategies scale data. The 50 data sets were factored. The mean eigenvalues for the first 8 consecutive components were 1.29 1.22, 1.18, 1.14, 1.10, 1.06, 1.02 and 1.006. Thus, only the first 1 eigenvalues change strategies scale factor analysis exceeded its associated eigenvalues derived from the random data and a 1-factor model was appropriate. This factor is accounted for 38.06% of the variability in the items. The internal consistency estimate for change strategies factors scale was substantial (alpha = 0. 78) and the test-retest reliability was also substantial (r = 0.74) (see Table 6).

Table 6: Factor analysis for physical activity change strategies scale (N = 512)

How often you do each of the following....	Factor loadings
I say positive things to myself about physical activity	0.71
I set goals to do physical activity	0.70
I do things to make physical activity more enjoyable	0.70
When I get off track with my physical activity plans, I tell myself I can start again and get right back on track	0.70
I keep track of how much physical activity I do	0.67
I reward myself for being physically active	0.66
I look for information about physical activity or sports	0.61
I try different kinds of physical activity so that I have more options to choose from	0.59
I make back-up plans to be sure I get my physical activity	0.59
I put reminders around my home to be physically active	0.57
I find ways to get around the things that get in the way of being physically active	0.56
I have a friend or family member who encourages me to do physical activity	0.55
I try to think more about the benefits of physical activity	0.53
I think about the benefits I will get from being physically active	0.52
I think about how my surroundings affect the amount of physical activity I do (Surroundings are things like having exercise equipment at home or a park near by)	0.46
Eigen value	5.7
% variance explained	38.6
Choronbach's alpha	0.78
Mean inter item correlation	0.34
Pearson test-retest*	0.74

*Test-retest stability with a 15-day interval (n = 93)

For the physical activity environmental factors, two subscales were also identified. The eigenvalues for the first 2 consecutive components were 2.03 and 1.009. Examination of the eigenvalues greater than 1 indicated that a 2-factor solution may be appropriate. The examination of the scree plot also suggested that 2 dimensions underlie environmental factors scale. In this study parallel analysis (PA) [36] was also employed to ascertain the optimal number of factors to extract. The mean eigenvalues for the first 3 consecutive components were 1.09 1.028 and 1.003. Thus, only the first 1 eigenvalues environmental factors scale factor analysis exceeded its associated eigenvalues derived from the random data and a 1-factor model was appropriate. This factor is accounted for 50.87% of the variability in the items. The internal consistency estimate for environmental factors scale was substantial (alpha = 67) and the test-retest reliability was moderate (r = 0.38) (see Table 7).

The findings showed intercorrelations among the physical activity-related psychosocial measures. Physical activity

Table 7: Factor analysis for physical activity environmental factors scale (N = 512)

	Factor loadings
It is safe to walk	0.78
Can get to easily	0.73
Enough supplies at Home	0.72
It is difficult to walk	0.60
Eigen value	2.03
% variance explained	50.87
Choronbach's alpha	0.67
Mean inter item correlation	0.25
Pearson test-retest*	0.38

* Test-retest stability with a 15-day interval (n = 93)

Table 8: Comparing Current study to Original study for reliability estimates of physical activity related psychosocial scales (n = 512)

		Current study	Original study
Variable	items	alpha	alpha
Change strategies	15	0.78	0.88
Self-efficacy	06	0.84	0.76
Pros	05	0.81	0.81
Cons	05	0.69	0.53
Family Support	04	0.72	0.79
Friend Support	05	0.77	0.60
Environment	04	0.67	0.42

self-efficacy was significantly and positively correlated with the physical activity pros scale (perceived benefits) and change strategies, while it was negatively correlated with the physical activity cons scale (perceived barrier).Those girls with higher scores on physical activity self-efficacy reported higher scores on physical activity pros, change strategies and lower scores on the physical activity cons.

Reliability

Reliability was determined by examining both the internal consistency and test-retest stability of the physical activity-related psychosocial measures. The physical activity-related psychosocial measures showed adequate internal consistency (i.e., > 0.70) [27] with the exception of the physical activity environmental factors which had an alpha of 0.67. However, this alpha is above the recommended lower level for group comparisons (i.e., > 0.50) [38]. As the physical activity environmental factors comprised 4 items the mean inter-item correlation is likely a more appropriate statistic for evaluating internal consistency. This measure, like coefficient alpha, produces an index of item homogeneity, but unlike the alpha is not affected by scale length [39]. For a reliable scale the mean inter item correlation should ideally be within the range of 0.20–0.40. However, values in the range of 0.10 to 0.50 are acceptable [39,40].

In addition, table 8 shows comparisons of psychometric properties of scores from the translated measures with those from the original measures [17].

Test-retest reliability

Almost 20% (93 subjects) of the original sample (512) were randomly selected to complete the physical activity-related psychosocial measures again 15 days after the initial assessment. Pearson Product Moment Correlations were calculated between the Time 1 and Time 2 assessments for the five scales. Results showed that the relationships were in the large effect size range for scales of physical activity self-efficacy, physical activity social support, physical activity pros and cons, physical activity change strategies and physical activity environmental factors, respectively (0.68, 0.55, 0.40, 0.74, and 0.38).

Discussion

The purpose of this study was to identify and evaluate the psychometric characteristics of physical activity-related psychosocial scales. This preliminary testing provides evidence for the reliability and validity of the physical activity- related psychosocial determinants questionnaires in Iranian high school girls.

An instrument containing the five scales was developed through a focus group with Iranian adolescent girls; the items were selected based on the consideration of contextually cultural relevance and language issues. Content validity of the instruments was established by having a panel of experts evaluating the instruments to obtain the most appropriate item content. The scales items were drawn from Norman studies [17] and confirmed by a focus group interview with the Iranian adolescent girls. The instruments were, then, refined based on expert judgment and exploratory factor analysis.

The obtained results from this study demonstrated acceptable internal consistency, good test-retest reliability and validity of the instruments in a large sample of Iranian adolescent girls. Of these five scales, four showed adequate internal consistency, using Chronbach alpha (i.e., > 70) [27], while the scale of environmental factors was lower in this regard (0.67). However, for this scale (which has 4 items) the mean inter-item correlation, a measure which is not affected by scale length, was acceptable (0.25). It would be useful for future researches to develop additional items for this scale. Chronbach's alpha values for the overall scales of physical activity self-efficacy, physical activity social support, physical activity pros and cons, physical activity change strategies, and physical activity

environmental factors ranged from 0.67 to 0.85. Test-retest reliability was also measured for overall the scales as ranging from fair to substantial (0.38–0.74). The notable exception was the environment scale. This is likely due to the nature of the items, which represent different domains of the environment such as sports equipment, neighbourhood recreation facilities, and neighbourhood safety. Because the items are not necessarily related to each other, internal consistency is not an appropriate indicator of scale quality.

A comparison of psychometric properties of scores from the translated measures with those from the original measures shows that both are similar, therefore, researches can use physical activity-related psychosocial scales to help promote physical activity levels among adolescents.

The exploratory factor analysis also identified subscales within the two of the five scales including: physical activity social support scale: family support and friend support, and physical activity decisional balance scale: pros and cons. The self-efficacy scale contained a single factor, the change strategies scale contained a single factor, and the environment scale also contained a single factor. Self-efficacy for physical activity had been used in previous studies and was kept as single dimensional scale. Also, social support and pros and cons for physical activity had been used in previous studies and thus were kept as multidimensional scales.

The most closely related previous study reported evidence in support of a one-dimensional scale of physical activity change strategies and scale of environmental factors [17,41], while our analysis suggest, the first, that physical activity change strategies scale and environmental factors scale are multidimensional, and next stage when used parallel analysis our result suggest that physical activity change strategies scale and environmental factors scale are one-dimensional.

These findings extend previous research by supporting single dimensional scale of physical activity self-efficacy [[17,41], and [42]]. However, Dwyer et al. suggested that physical activity self-efficacy is multidimensional: self-efficacy to overcome external barriers and self-efficacy to overcome internal barriers [43]. This difference may be due to this fact that there are some cultural barriers to Iranian girls exercising in public places. There are only few girl fitness centres, which few can afford.

Intercorrelations between the physical activity-related psychosocial scales were fair to moderate suggesting that psychosocial sub-scales are generally independent.

The analysis reported here provides further empirical support for the relevance of Bandura's social cognitive theory to the studies of the psychosocial determinants of physical activity.

In spite of the suitable design and use of exploratory factor analysis in this study, several limitations were noted. First, there are no theoretically based questionnaires of physical activity related psychosocial determinants for adolescents in Iran. Second, since our sample consisted of adolescents from a specific education area in Tehran, our results could not be generalized to adolescents who live in other geographic locations in Iran. Therefore, future research should replicate this study with a sample of adolescents living in others education areas.

Conclusion

In summary, development of questionnaires to measure physical activity- related psychosocial determinants in Iranian adolescents is still in its developmental stage. These measures warrant further study to strengthen their measurement properties, but may be useful in future studies for examining the factors that contribute to physical activity in Iranian adolescent girls. We believe the behavior change construct measures that demonstrated strong psychometric properties will be useful instruments for measuring adolescents in observational and experimental studies of physical activity. Further work is needed to refine the measures that need to be improved and to assess the construct validity of these measures.

In conclusion, the results of this study provide evidence for the soundness of factor structure and acceptable reliability of the scales of physical activity- related psychosocial determinants in the Iranian population.

Competing interests

The authors declare that they have no competing interests.

Authors' contributions

AP, AH, SF, FG and AA contributed equally to the design and conduct of the survey, analysis of the results, drafting and critical revision of the manuscript. AP, AH, AA, SF and FG read and approved the final version of the manuscript.

Additional material

Additional file 1

Final translation of psychosocial determinants of physical activity questionnaires. Translation and back translation procedure was used to develop culturally equivalent questionnaires. ***Self – efficacy of physical activity****. There are many barriers in the way of physical activity. Mark the extent of your capability for doing physical activity in the following situations. Answer all the questions. 1. Can you do exercise or physical activity even when you are sad or under stress? 2. Can you dedicate a specific time for doing exercise or physical activity on most days of the week?. 3. Can you do exercise or physical activity even when your family or friends ask you to do something else?. 4. Can you get up early to do your exercise or physical activity even on the weekends?. 5. Can you do your exercise or physical activity even when you have a lot of homework to do?. 6. Can you do your exercise or physical activity even when the weather is hot or it is rainy?.* ***Family support of physical activity****. 1. How many days a week do your family members watch your exercise or physical activity?. 2. How many days a week do your family members encourage you to do exercise or physical activity?. 3. How many days a week do your family members provide you transportation go to a place for doing exercise or physical activity?. 4. How many days a week do your family members do their exercise or physical activity with you?.* ***Friend support of physical activity****. 1. How many days a week do your friends encourage you to do exercise or physical activity?. 2. How many days a week do your friends do their exercise or physical activity with you?. 3. How many days a week do your friends tease you for doing well your exercise or physical activity?. 4. How many days a week do your friends ask you to walk or bike from your house to the school or their houses?. 5. How many days a week do your friends tell you that you are doing well your exercise or physical activity?. 6. How many of your 5 closest friends do their physical activity regularly?.* ***Positive and negative of physical activity****. The following sentences are different beliefs about physical activity. Please choose the degree which indicates the importance of each sentence to you whenever deciding whether or not to do your physical activity. 1. I would feel embarrassed if people saw me doing physical activity. 2. Physical activity helps me to keep fit. 3. My parents would become happy if I do physical activity. 4. I have to learn a lot of things to be able to do physical activity. 5. If I do physical activity I would feel better about myself. 6. I would need too much help from my parents to be able to do physical activity. 7. Physical activity and exercise makes me unpleasant feeling. 8. I would have fun doing physical activity or playing sports with my friends. 9. If I do physical activity I would have more energy. 10. Physical activity reduces the time I spend for being with my friends.* ***The strategies to change physical activity****. The followings are activities/thoughts, and feelings people use to help them change their physical activity. Think about yourself similar cases that you experience or have experienced during the past month then indicate HOW OFTEN you do each of the followings:. 1. I look for information about physical activity or sports. 2. I keep the account of my physical activity. 3. I find out the ways to overcome the obstacles of doing physical activity. 4. I think about the effect of facilities (such as having sports equipments at home or in the near by park) on the amount of my physical activity. 5. To be more physically active I keep things at home to remind me that. 6. I encourage myself for doing physical activity or exercise. 7. I do things to make physical activity more enjoyable. 8. I think about the benefits I get from the physical activity or exercise. 9. I think about the benefits of physical activity than its troubles. 10. I say to myself about the positive sides of physical activity. 11. When my physical activity plans are stopped for a while,. I tell myself I can start again and become active. 12. I have a friend or family member who encourages me to do physical activity. 13. I try different kinds of physical activity to have more options to choose from. 14. I set goals for doing physical activity. 15. I consider alternative plans to assure myself of having physical activity.* ***Environmental factors of physical activity:****. Define your attitude about your life environment and indicate how much do you agree with the following sentences?. 1. There is enough sports equipment at home to use for physical activity. 2. Walking or jogging is difficult around my house, because of traffic, lack of side walks, dogs, gangs, and so on. 3. It is possible to access playgrounds, parks or gyms near to my home for doing physical activity or exercise. 4. It is safe to walk or jog during the day around my home.* ***Physical activity enjoyment****. 1. I enjoy doing physical activity or exercise.* ***Recreation choices of physical activity****. 2. Which of the following activities do you usually choose to spend on your leisure time?. (1) I almost always choose the activities like watching T.V, studying, listening to music, or working with computer. (2)I usually most always choose the activities like watching T.V, studying, listening to music, or working with computer. (3) It is possible to choose active entertainments as much as inactive ones. (4) I usually choose the activities like riding, bike, skating, and games played, outside or active sports. (5) I almost always choose the activities like riding, bike, skating, and games played, outside or active sports.*

Click here for file
[http://www.biomedcentral.com/content/supplementary/1471-2458-8-150-S1.doc]

Acknowledgements

The authors would like to gratefully acknowledge Prof. Greg Norman and Prof. Sallis James for their permission to use the PACE-Adolescent Physical Activity Survey in this study. We would like to acknowledge, with gratitude, financial support from Tarbiat Modares University.

References

1. US Public Health Service Office of the Surgeon General: **Physical Activity and health.** *a report of the Surgeon General. Atlanta, GA, [Washington, DC], Pittsburgh, PA: US Department of Health and Human Services, Centers for Disease Control and Prevention, National Center for Chronic Disease Prevention and Health Promotion; President's Council on Physical Fitness and Sports* 1996.
2. Telama K, Yang X, Uakso L, Viikari J: **Physical activity in childhood and adolescents as predictors of physical activity in young adulthood.** *American Journal of Preventive Medicine* 1997, **13:**317-323.
3. Sallis JF: **Age-related decline in physical activity: a synthesis of human and animal studies.** *Medicine and Science in Sports and Exercise* 2000, **32(9):**1598-1600.
4. Dishman RK: **Predicting and changing exercise and physical activity: What's practical and what's not. In.** In *Toward active living* Edited by: Quinn H, Gauvin L, Wall A. Champaign (IL): Human Kinetics; 1994:97-106.
5. Sheikholeslam R, Mohamad A, Mohammad K, Vaseghi S: **Non-communicable disease risk factors in Iran.** *Asia Pac J Clinical Nutrition* 2004, **13(Suppl 2):**S100.
6. Kelishadi R, Sadri GH, Tavasoli AA, Kahbazi M, Roohafza HR, Sadeghi M: **The cumulative prevalence of atherosclerotic cardiovascular diseases' risk factors in Iranian adolescents.** *Journal Pediatric* 2005, **81:**447-53.
7. Marcus BH, Banspach SW, Lefebvre RC, Rossi JS, Carleton RA, Abrams DB: **Using the stages of change model to increase the adoption of physical activity among community participants.** *American Journal Health Promotion* 1992, **6(6):**424-9.
8. Buckworth J, Dishman RK: **Determinants of physical activity: research to application.** In *Lifestyle Medicine* Edited by: Rippe J. Malden, MA: Blackwell Science; 1999:1016-1027.
9. Bandura A: **Social foundations of thought and action: a social cognitive theory.** Englewood Cliffs, (NJ), Prentice Hall; 1986.
10. Prochaska JO, DiClement CC: **Stages and processes of self-change of smoking: Toward an integrative model of change.** *Journal of Consulting and Clinical Psychology* 1983, **51:**390-395.
11. Washington DC, Pittsburgh PA: *US Department of Health and Human Services, Centers for Disease Control and Prevention, National Center for Chronic Disease Prevention and Health Promotion; President's Council on Physical Fitness and Sports* 1996.
12. Prochaska JO, Velicer WF: **The transtheoretical model of health behavior change.** *Am J Health Prom* 1997, **12:**38-48.
13. Garner C, Page SJ: **Applying the transtheoretical model to the exercise behaviors of stroke patients.** *Top stroke Rehabit* 2005, **12(1):**69-75.
14. Kline P: **An Easy Guide to Factor Analysis.** Routledge, London; 1994.
15. Messick S: **Validity of psychological assessment: validation of inferences from persons' responses and performances as scientific inquiry into score meaning.** *American Psychologist* 1995, **50(9):**741-749.
16. Dixon JK: **Factor analysis. In: Munro BH. Statistical Methods for Health Care Research.** 4th edition. New York: Lippincott; 2001:303-329.
17. Norman GJ, Sallis JF, Gaskins R: **Comparability and reliability of paper- and computer-based measures of psychosocial constructs for adolescent physical activity and sedentary behaviors.** *Res Q Exerc Sport* 2005, **76(3):**315-23.
18. Brislin RW: **Back-translation for cross-cultural.** *Psychology* 1970, **31(3):**185-216.
19. Jones EG, Kay M: **Instrumentation in cross-cultural research.** *Nursing Research* 1992, **41(3):**186-188.
20. Sallis JF, Pinski RB, Grossman RM, Patterson TL, Nader PR: **The development of self-efficacy scales for health-related diet and exercise behaviors.** *Health Education Research* 1988, **3:**283-292.
21. Marcus BH, Owen N: **Motivational readiness, self-efficacy and decision-making for exercise.** *Journal of Applied Social Psychology* 1992, **22:**3-16.

22. Marcus BH, Rakowski W, Rossi JS: **Assessing motivational readiness and decision making for exercise.** *Health Psychol* 1992, **11:**257-261.
23. Sallis JF, Taylor WC, Dowda M, Freedson PS, Pate RR: **Correlates of vigorous physical activity for children in grades 1 through 12: Comparing parent-reported and objectively measured physical activity.** *Pediatric Exercise Science* 2002, **14:**30-44.
24. Marcus BH, Rossi JS, Selby VC, Niaura RS, Abrams DB: **The stages and processes of exercise adoption and maintenance in a worksite sample.** *Health Psychol* 1992, **11:**386-395.
25. Saelens BE, Gehrman CA, Sallis JF, Calfas KJ, Sarkin JA, Caparosa S: **Use of self-management strategies in a 2-year cognitive-behavioral intervention to promote physical activity.** *Behavior Therapy* 2000, **31:**365-379.
26. DeVellis RF: **Scale development.** In *theory and applications* New York: Sage; 1991.
27. Nunnally JC, Bernstein IH: 3rd edition. New York: McGraw-Hill; 1994.
28. Landis JR, Koch GG: **The measurement of observer agreement for categorical data.** *Biometrics* 1977, **33(1):**159-74.
29. Rummel RJ: *Applied factor analysis* Evanston (IL): Northwestern Univ. Press; 1970.
30. Child D: **The Essentials of Factor Analysis.** Cassel, London; 1990.
31. Tabachnick BG, Fidell LS: **Using Multivariate Statistics.** 3rd edition. New York: HarperCollins; 1996.
32. Bartlett MS: **A note on the multiplying factors for various chi square approximations.** 1954.
33. Cattell RB: **The scree test for the number of factors.** *Multivariate Behavioral research* 1966, **1:**245-276.
34. Ferguson E, Cox T: **Exploratory factor analysis: a users' guide.** *International Journal of Selection and Assessment* 1993, **1:**84-94.
35. Zwick WR, Velicer WF: **Comparison of five rules for determining the number of components to retain.** *Psychological Bulletin* 1986, **99:**432-442.
36. Horn JL: **A rational and test for the number of factor analysis.** *Psychometrika* 1965, **30:**179-185.
37. Thompson B, Daniel LG: **Factor analytic evidence for the construct validity of scores: a historical overview and some guidelines.** *Educational and Psychological Measurement* 1996, **56:**197-208.
38. Helmstadter GC: **Principles of Psychological Measurements.** New York: Appleton-Century-Crofts; 1964.
39. Cox T, Ferguson E: **Measurement of the subjective work environment.** *Work and Stress* 1994, **8:**98-109.
40. Ferguson E, Daniel E: **The Illness Attitudes Scale (IAS): A psychometric evaluation on a non-clinical population.** *Personality and Individual Differences* 1995, **18:**463-469.
41. Motl RW, Dishman RK, Ward DS, Saunders RP: **Perceived physical environment and physical activity across one year among adolescent girls: self-efficacy as a possible mediator?** *J Adolescent Health* 2005, **37(5):**403-408.
42. Sherwood NE, Taylor WC, Treuth M, Klesges LM, Baranowski T, Zhou A, Pratt C, McClanahan B, Robinson TN, Pruitt L, Miller W: **Measurement characteristics of activity-related psychosocial measures in 8- to 10-year-old African-American girls in the Girls Health Enrichment Multisite Study (GEMS).** *Preventive Medicin* 2004, **38:**S60-8.
43. Dwyer JJ, Allison KR, Makin S: **Internal structure of a measure of self-efficacy in physical activity among high school students.** *Social science medicine* 1998, **46(9):**1275-82.

Pre-publication history

The pre-publication history for this paper can be accessed here:

http://www.biomedcentral.com/1471-2458/8/150/prepub

Publish with **BioMed Central** and every scientist can read your work free of charge

"BioMed Central will be the most significant development for disseminating the results of biomedical research in our lifetime."

Sir Paul Nurse, Cancer Research UK

Your research papers will be:

- available free of charge to the entire biomedical community
- peer reviewed and published immediately upon acceptance
- cited in PubMed and archived on PubMed Central
- yours — you keep the copyright

Submit your manuscript here:
http://www.biomedcentral.com/info/publishing_adv.asp

BioMedcentral
