# Supplementary material for: Associations between parental support and physical activity in children and adolescents with visual impairments: a mediating analysis of exercise self-efficacy
Source: PeerJ. 2026 Oct 28;14:e21871. doi: 10.7717/peerj.21871 (PMC13723195; doi:10.7717/peerj.21871)
Supplement: Supplementary file 3 [file peerj-14-21871-s009.pdf]

This article was downloaded by: [Central Michigan University]
On: 01 December 2014, At: 09:56
Publisher: Routledge
Informa Ltd Registered in England and Wales Registered Number: 1072954 Registered office: Mortimer House, 37-41 Mortimer Street, London W1T 3JH, UK

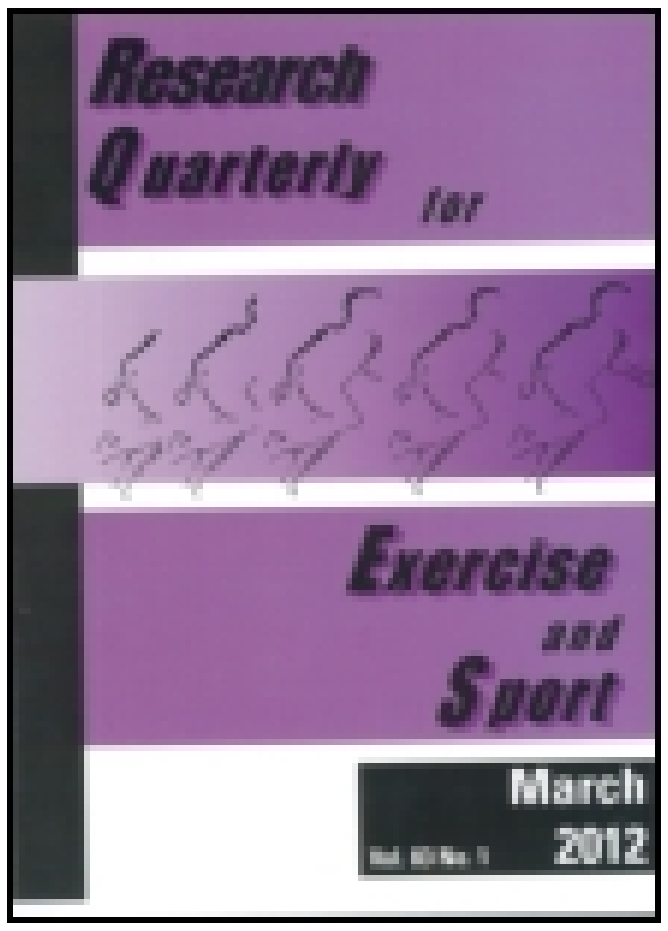

Research Quarterly for Exercise and Sport
Publication details, including instructions for authors and subscription information:
http://www.tandfonline.com/loi/urqe20

Association of Parent and Peer Support with Adolescent Physical Activity

Judith J. Prochaska [a], Miki W. Rodgers [b] & James F. Sallis [b]

[a] Joint Doctoral Program in Clinical Psychology, San Diego State University and the University of California-San Diego, USA

[b] Department of Psychology, San Diego State University, USA

Published online: 26 Feb 2013.

To cite this article: Judith J. Prochaska, Miki W. Rodgers & James F. Sallis (2002) Association of Parent and Peer Support with Adolescent Physical Activity, Research Quarterly for Exercise and Sport, 73:2, 206-210, DOI: 10.1080/02701367.2002.10609010

To link to this article: http://dx.doi.org/10.1080/02701367.2002.10609010

PLEASE SCROLL DOWN FOR ARTICLE

Taylor & Francis makes every effort to ensure the accuracy of all the information (the “Content”) contained in the publications on our platform. However, Taylor & Francis, our agents, and our licensors make no representations or warranties whatsoever as to the accuracy, completeness, or suitability for any purpose of the Content. Any opinions and views expressed in this publication are the opinions and views of the authors, and are not the views of or endorsed by Taylor & Francis. The accuracy of the Content should not be relied upon and should be independently verified with primary sources of information. Taylor and Francis shall not be liable for any losses, actions, claims, proceedings, demands, costs, expenses, damages, and other liabilities whatsoever or howsoever caused arising directly or indirectly in connection with, in relation to or arising out of the use of the Content.

This article may be used for research, teaching, and private study purposes. Any substantial or systematic reproduction, redistribution, reselling, loan, sub-licensing, systematic supply, or distribution in any form to anyone is expressly forbidden. Terms & Conditions of access and use can be found at http://www.tandfonline.com/page/terms-and-conditions

Research Quarterly for Exercise and Sport
©2002 by the American Alliance for Health,
Physical Education, Recreation and Dance
Vol. 73, No. 2, pp. 206–210

Association of Parent and Peer Support With Adolescent Physical Activity

Judith J. Prochaska, Miki W. Rodgers, and James F. Sallis

Downloaded by [Central Michigan University] at 09:56 01 December 2014

Key words: determinants, exercise, social support, youth

The Council on Physical Education for Children (1998) and an international consensus group recommend that young people accumulate 60 min of moderate or greater intensity activity on most days of the week (Cavill, Biddle, & Sallis, 2001). A substantial proportion of adolescents, in particular girls and ethnic minorities, are not meeting physical activity recommendations (Centers for Disease Control and Prevention, 2000). Research is needed to determine the factors that influence physical activity participation in this age group.

The current study focuses on the relationship of social support to youth physical activity. Social support for physical activity can take many forms. It may be instrumental and direct (transportation, payment of fees), emotional and motivational (encouragement, praise), or observational (modeling). In early childhood, parents and siblings serve as the most important behavioral role models. While parents continue to influence adolescents' health behaviors, the role of peer influence is believed to increase with age (Eccles, 1999). Peers exert their influence on adolescent behavior through imitation and social reinforcement (Wold & Hendry, 1998).

In the literature, the association between social support and youth physical activity is inconsistent. Previously we reviewed the determinants of physical activity for children and adolescents (Sallis, Prochaska, & Taylor, 2000). Eighteen studies examined the association of social support to physical activity. The inconsistency in findings was attributed to different measures of social support that varied in reliability and validity and different modes of physical activity measurement. In only two studies were the social support measures evaluated for reliability (test-retest); none of the studies reported evidence of validity (i.e., field test or readability statistics). Four studies used objective measures of physical activity (e.g., accelerometer, observation); the remainder ($n = 14$) relied solely on self-report. Studies reported multiple tests of the association between physical activity and social support. Of 116 tests reported, 51 (44%) indicated a significant association. Within studies, those that relied on self-reported physical activity were more likely to find a significant association ($M = 57\%$ of tests), than studies that used objective measures ($M = 25\%$ of tests). For adolescents, it also may be important to measure the influence of parent and peer support separately.

The purpose of the current study was to determine if the association between social support and adolescent physical activity would vary, when different methods of assessing physical activity were used and when social support was separated into parent and peer social support. Physical activity was measured with an accelerometer and by self-report.

Submitted: September 26, 2000
Accepted: June 28, 2001

Judith Prochaska is with the Joint Doctoral Program in Clinical Psychology at San Diego State University and the University of California–San Diego. Miki W. Rodgers and James F. Sallis are with the Department of Psychology at San Diego State University.

Method

Sample

Data analyzed in the current study were collected as part of the PACE+ School Study, a randomized controlled evaluation of a computer-based physical activity and nutrition intervention (Prochaska et al., 2000).

Downloaded by [Central Michigan University] at 09:56 01 December 2014

Participants were recruited from a public suburban middle school (grades 6 to 8) in San Diego, CA. School enrollment was 1,111; 24% of the student body received subsidized lunches (an indicator of school socioeconomic level). Participants were recruited from required courses (e.g., life science, physical education). Criteria for participation included ability to read English, engage in physical activity (e.g., no cardiovascular or musculoskeletal problems), parental consent, and child assent. The study protocol received human subjects approval. Ostensibly, the entire student body was exposed to recruitment efforts through bulletins in the school newsletter and presentations in physical education classes. The extent to which individual students were exposed to recruitment information, however, is not known. A total of 138 students participated (12% participation rate).

Measures

Computer Science and Applications (CSA) Activity Monitor. The CSA is a small, durable, and lightweight accelerometer (Computer Science and Applications, Inc., Shalimar, FL) that measures movement in the vertical plane. The CSA measures the duration, frequency, and intensity of physical activity using a 1-min sampling interval. In the current study, participants were assigned a CSA on a belt and instructed to wear it over their right hip at all times for a 5-day period, except when sleeping, showering, and swimming. In laboratory and field settings, the CSA has been shown to be a valid tool for quantifying children's activity levels (Trost et al., 1998).

PACE+ Physical Activity Screener. The PACE+ screener (two items) is a paper-based self-report survey that assessed number of days the adolescents participated in physical activity for 60 min or more over the previous 7 days and for a typical week (Prochaska, Sallis, & Long, 2001). The measure broadly defined physical activity as that which "increases your heart rate and makes you get out of breath some of the time" and did not specify intensity. Examples of physical activities were provided (e.g., brisk walking, soccer). Directions instructed respondents to add the minutes of physical activity accumulated over the day and only count days they were physically active for 60 min or more. A composite average of the two items yielded a score of days per week (range: 0–7) the adolescent accumulated 60 min of moderate-to-vigorous physical activity. Prior evaluation of the measure demonstrated high test-retest reliability ($ICC = .79$) and significant correlation with CSA data. In the current study, participants completed the PACE+ screener after wearing the CSA; thus, reports of physical activity for the previous 7 days reflected the same assessment period. The measures correlated significantly ($r = .46, p < .01$).

Parent Support. The parent support scale was developed for the Amherst Health and Activity Study (Sallis, Taylor, Dowda, Freedson, & Pate, in press). The 5-item parent support scale was completed by the adolescent and assessed parental: (a) encouragement, (b) praise, (c) transportation of the adolescent to physical activity settings, (d) physical activity participation with the adolescent, and (e) watching the adolescent's participation in physical activity or sports. Frequency of social support behaviors was rated on a 6-point Likert-type scale ranging from never (coded 0) to everyday (coded 5). Total scores could range from 0 to 25. The scale was piloted with 63 parent-child pairs (Sallis et al., in press). Two-week test-retest reliability of the scale was strong ($ICC = .88$), and parent and child reports correlated significantly ($r = .61, p < .001$). Internal consistency of the items evaluated with the current sample was $\alpha = .77$.

Peer Support. The 4-item peer support scale was developed for the Amherst Study and was completed by the adolescent to assess peer: (a) encouragement, (b) participation, (c) praise, and (d) the adolescent's encouragement of peers to be physically active. Frequency of support behaviors was rated on a 6-point Likert-type scale ranging from never (coded 0) to everyday (coded 5). Total scores could range from 0 to 20. The original scale included an item that assessed peer teasing related to physical activity (reverse coded). In a pilot study, 2-week test-retest reliability was strong ($ICC = .86$), and parent and child reports correlated significantly ($r = .57, p < .001$) (Sallis et al., in press). In the current study, internal consistency was reduced with the teasing variable ($\alpha = .54$) but enhanced with an item assessing peers' praise ($\alpha = .81$). The peer praise item was used in place of peer teasing.

Analysis

CSA data were analyzed using a software program developed by Trost et al. (1998) that calculated total minutes spent in moderate (3 to 5.99 METs), vigorous (6 to 8.99 METs), and very vigorous (≥ 9 METs) physical activity for each day assessed. One MET is the metabolic equivalent to an individual sitting at rest. CSA data were calibrated based on laboratory measurement of oxygen consumption during treadmill locomotion with youth 6–18 years old (LaRoche, Debold, Sirard, Hendel-man, & Freedson, 1998). The equation adjusted for age and gender. Days with less than 8 hr of activity counts were considered missing. All participants were included in the analyses; the days of CSA data varied from 2 to 5. The CSA outcome variable was the average minutes per day of moderate-to-vigorous physical activity. The data were entered into SPSS Version 9.0 for analyses. Associations with social support were tested separately with self-reported and monitored physical activity. A standardized composite of physical activity was created to examine the benefit of combining the two assessment modes into a

single index. The self-report and monitored physical activity values were standardized (i.e., *z* score) and then summed.

Descriptive statistics summarized the sample's demographic characteristics. Correlations tested the associations between social support (parent and peer) and the three physical activity measures (self-report, monitor, standardized composite). Multiple associations were examined, and 2-tailed tests were used to be conservative. Hierarchical multiple regressions examined the association of parent and peer support with the physical activity measures, after controlling for gender, grade, and race. Due to small numbers for some ethnic groups, race was recoded to compare Caucasians, Asian Pacific Islanders, and other racial groups. The demographic variables were entered first as a block, followed by a block with the social support scales. The total adjusted multiple correlation (R^2) indicated the proportion of variance in physical activity levels predicted by the full model. Change in the adjusted R^2 indicated the amount of variance explained by each block. For each variable, the partial correlation indicated the contribution of that variable to predict the model, after adjusting for all other variables.

Results

Descriptive Statistics

The sample (N = 138) had a mean age of 12.1 years (SD = .9). Ethnic distribution was 28% Caucasian, 23% Asian Pacific Islander, 7% African American, 5% Latino, and 37% other (e.g., Native American, multiracial). Of the sample, 65% were girls, with 48 participants in the sixth grade (52% girls), 39 in the seventh grade (62% girls), and 51 in the eighth grade (80% girls). The sample reported a mean of 4.8 days per week (SD = 2.0) on the PACE+ screener. Participants averaged 84.9 min (SD = 31.3) on the CSA. Mean total scores on the parent and peer support scales were 10.1 (SD = 4.6) and 8.6 (SD = 3.7), respectively.

Correlations

The parent and peer full scales and eight of the nine individual items correlated significantly with self-reported physical activity (see Table 1). In contrast, neither full scale nor any of the individual items correlated significantly with monitored physical activity. For six of the items, correlations approached zero ($r \leq |.05|$). Correlations with the standardized composite were stronger than for monitored physical activity but weaker than those for self-report.

Hierarchical Regressions

For self-reported physical activity, the demographic block accounted for 7% of the variance (see Table 2). Gender was the only significant correlate, with boys reporting greater physical activity than girls ($p < .05$). The social support block contributed an additional 12%. Peer support was the strongest correlate for self-reported physical activity (partial r = .24). For monitored physical activity, the demographic block accounted for 14% of the variance. Gender and race were significant corre-

Table 1. Correlations of support behaviors with self-reported and monitored physical activity and a standardized composite

Source	Support behavior	Self-report	Monitor	Composite[1]
Parent				
Full scale (α = .77)		.31**	.12	.27**
Parent encourages adolescent to do physical activity or sports		.08	.07	.10
Parent does physical activity or sports with adolescent		.24**	.08	.20*
Parent provides transportation to physical activity setting		.28**	.02	.19*
Parent watches adolescent do physical activity or sports		.32**	.16	.30**
Parent tells adolescent she or he is doing well in physical activity or sports		.24**	.12	.22**
Peer				
Full scale (α = .81)		.35**	.01	.22**
Adolescent encourages friends to do physical activity or sports		.34**	-.10	.21*
Friends encourage adolescent to do physical activity or sports		.29**	-.05	.15
Friends do physical activity or sports with adolescent		.22*	.05	.17
Friends tell adolescent she or he is doing well in physical activity or sports		.27*	.05	.20*

[1]Sum of standardized (*z* score) values for self-report and monitored PA measures.
*p <.05 (2-tailed).
**p < .01 (2-tailed).

Downloaded by [Central Michigan University] at 09:56 01 December 2014

Downloaded by [Central Michigan University] at 09:56 01 December 2014

lates, with girls and Asian Pacific Islanders having fewer minutes of monitored physical activity than boys and participants of other races ($p < .01$). The social support block did not contribute significantly to the model. Findings for the standardized composite seemed to be an average of the findings for self-report and monitored physical activity. The social support block contributed significantly (4%) to the model after controlling for demographic factors, but neither scale was a significant correlate alone.

Discussion

In examining the association between social support and adolescent physical activity, this study sought to demonstrate that the association would vary depending on the physical activity measure and on separating social support into peer and parental influences. Mirroring the literature, the findings were inconsistent and suggestive of possible methodological influences. In the current study, parent and peer support significantly correlated with adolescent self-reported physical activity. The association was significant for eight of the nine individual social support items. In a hierarchical regression model, peer support was the strongest correlate of self-reported physical activity after controlling for gender, grade, and race. Correlations with monitored physical activity, however, were all nonsignificant, and the social support scales did not contribute significantly in a hierarchical model. Combining the two modes of physical activity measurement in a standardized composite did not strengthen associations with social support beyond that observed with self-report alone. The association between social support and adolescent physical activity was consistent across sources (parent and peer) but inconsistent across modes of physical activity assessment (self-report and monitor).

In their study with adults, Dishman, Darracott, and Lambert (1992) demonstrated a similar discrepancy in the significance of theoretical determinants based on the method used to assess physical activity. Psychological variables that significantly correlated with self-report were unrelated to objective monitoring with a Caltrac (Hemokinetics, Madison, WI) motion sensor. Research on the determinants of youth physical activity has relied predominantly on self-report measures (Sallis et al., 2000). Researchers, however, have questioned the validity and generalizability of findings based solely on self-report and concluded that the study of physical activity determinants has been weakened because of reliance on less precise measures (e.g., Dishman & Sallis, 1994). The current study and research by Dishman et al. (1992), however, indicate that associations are weakened rather than strengthened when objective, and presumably more valid, measures are used. A possible explanation is that shared method variance led to overestimation of the correlation. The association between the social support scales and self-reported physical activity may be due to the measures' common mode of assessment (i.e., self-report).

Another explanation for the lack of correlation with the CSA may be that monitors pick up incidental physical activity throughout the day that may not be influenced by theoretically based psychosocial variables. In contrast, the social support scales and physical activity self-report measure referred specifically to intentional

Table 2. Hierarchical regression analyses explaining self-reported and monitored physical activity and a standardized composite

Block of variables	Self-reported physical activity		Monitored physical activity		Composite[a] physical activity	
	t	Partial r	t	Partial r	t	Partial r
Demographics	Adjusted-R^2 = .072*		Adjusted-R^2 = .143***		Adjusted-R^2 = .170***	
Gender (girls)	-2.42*	-.21	-3.33**	-.28	-3.71***	-.31
Grade (sixth)	.63	.06	1.74	.15	1.53	.13
Grade (seventh)	-1.62	-.14	.77	.07	-.52	-.05
Race (Caucasian)	-1.26	-.02	-.99	-.08	-3.21**	-.27
Race (Asian Pacific Islander)	-1.90	-.17	-3.07**	-.26	-1.41	-.12
Social support	Δ Adjusted-R^2 = .115***		Δ Adjusted-R^2 = -.006		Δ Adjusted-R^2 = .044*	
Parent support	1.51	.13	1.11	.09	1.65	.14
Peer support	2.77**	.24	-.72	-.06	1.28	.11
Full model	Total adjusted-R^2 = .187***		Total adjusted-R^2 = .137***		Total adjusted-R^2 = .214***	

[a]Sum of standardized (z score) values for self-report and monitored physical activity measures.
*$p < .05$.
**$p < .01$.
***$p < .001$.

Downloaded by [Central Michigan University] at 09:56 01 December 2014

physical activity and sports. At the same time, the CSA is known to miss some types of activities (Trost et al., 1998), so this limitation could reduce observed correlations. Dishman et al. (1992) suggested the use of multimodal measures (monitor, self-report, observation) to better characterize free-living physical activity. In the current study, combining CSA and self-report data in a composite index reduced associations with social support. The strengths of this study included use of reliable measures and separate assessment of parent and peer support. The high internal consistency of the support scales indicated that either the adolescents were not discriminating among the items or that support behaviors are actually strongly clustered. The social support scales assessed the behaviors most commonly studied in the literature (encouragement, transportation, modeling) and several others (parent watching, praise, encouragement of friends).

Conclusion

Is social support related to adolescent physical activity? Unfortunately, the findings are still inconclusive, although the current study suggests some potential explanations for the discrepancy in the literature. For self-reported physical activity, peer and parent support were significant correlates, and, in a hierarchical regression model, peer support remained significant after controlling for demographic variables. Although shared method variance likely accounts for some of the association observed, perceived social support appears to be associated with perceived engagement in physical activity. Disappearance of the association when using an objective measure raises the possibility that more global activity levels are unrelated to social support. The current findings identify the need to incorporate multiple modes of measurement in future studies of the determinants of youth physical activity.

References

Cavill, N., Biddle, S., & Sallis, J. (2001). Health enhancing physical activity for young people: Statement of the United Kingdom expert consensus conference. *Pediatric Exercise Science, 13*, 12–25.

Centers for Disease Control and Prevention. (2000). Youth risk behavior surveillance—United States, 1999. *Morbidity & Mortality Weekly Report, 49*, 1–96.

Council on Physical Education for Children. (1998). *Physical activity for children: A statement of guidelines.* Reston, VA: National Association for Sport and Physical Education.

Dishman, R. K., Darracott, C. R., & Lambert, L. T. (1992). Failure to generalize determinants of self-reported physical activity to a motion sensor. *Medicine and Science in Sports and Exercise, 24*, 904–910.

Dishman, R. K., & Sallis, J. F. (1994). Determinants and interventions for physical activity and exercise. In C. Bouchard, R. J. Shephard, & T. Stephens (Eds.), *Physical activity, fitness, and health* (pp. 214–238). Champaign, IL: Human Kinetics Publishers

Eccles, J. S. (1999). The development of children ages 6 to 14. *The Future of Children, 9*, 30–44.

LaRoche, D. P., Debold, E. P., Sirard, J. R., Hendelman, D. L., & Freedson, P. S. (1998). Prediction of oxygen consumption during treadmill exercise in children and adolescents (Abstract). *Medicine and Science in Sports and Exercise, 30*(Suppl), 152.

Prochaska, J. J., Sallis, J. F., Cohen, D., Schmid, B., Watanabe, M., & Patrick, K. (2000). Acceptability of a computer-based intervention promoting physical activity & fruits and vegetables with middle school students (Abstract). *International Journal of Behavioral Medicine, 7*(Suppl 1), 107.

Prochaska, J. J., Sallis, J. F., & Long, B. (2001). A physical activity screening measure for use with adolescents in primary care. *Archives of Pediatrics and Adolescent Medicine, 155*, 554–559.

Sallis, J. F., Prochaska, J. J., & Taylor, W. C. (2000). A review of correlates of physical activity of children and adolescents. *Medicine and Science in Sports and Exercise, 32*, 963–975.

Sallis, J. F., Taylor, W. C., Dowda, M., Freedson, P. S., & Pate, R. R. (2002). Correlates of vigorous physical activity for children in grades 1 through 12: Comparing parent-reported and objectively monitored physical activity. *Pediatric Exercise Science, 14*, 30–44.

Trost, S. G., Ward, D. S., Moorehead, S. M., Watson, P. D., Riner, W., & Burke J. R. (1998). Validity of the computer science and applications (CSA) activity monitor in children. *Medicine and Science in Sports and Exercise, 30*, 629–633.

Wold, B., & Hendry, L. (1998). Social and environmental factors associated with physical activity in young people. In S. Biddle, J. Sallis, & N. Cavill (Eds.), *Young and active? Young people and health-enhancing physical activity—evidence and implications* (pp. 119–132). London: Health Education Authority.

Authors' Notes

Data collection was supported by a Predoctoral Psychosocial Grant from the American Cancer Society, California Division, and a Dissertation Grant from the American College of Sports Medicine. Please address all correspondence concerning this article to Judith J. Prochaska, University of California–San Francisco, 401 Parnassus Avenue-CPT, San Francisco, CA 94143-0984.

E-mail: prochask@sunstroke.sdsu.edu.
