# Supplementary material for: Associations between parental support and physical activity in children and adolescents with visual impairments: a mediating analysis of exercise self-efficacy
Source: PeerJ. 2026 Oct 28;14:e21871. doi: 10.7717/peerj.21871 (PMC13723195; doi:10.7717/peerj.21871)
Supplement: Supplementary file 5 [file peerj-14-21871-s003.docx]

|  | | Item No | Recommendation |
| --- | --- | --- | --- |
| **Title and abstract** | | 1 | (*a*) Cross-sectional study **(page1)** |
|  |  |  | (*b*) This paper aimed to examine the associations between parental support and moderate-to-vigorous physical activity (MVPA) levels in children and adolescents with visual impairment (VI), as well as the mediating role of exercise self-efficacy. Significant positive correlations were observed between parental support and MVPA (r=0.306, P<0.01), as well as between exercise self-efficacy and MVPA (r=0.385, P<0.01). Exercise self-efficacy significantly mediated the relationship between parental support and MVPA (indirect effect =0.085, 95% CI [0.039, 0.146]), accounting for 26.3% of the total effect. |
| Introduction (page3-6) | | | |
| Background/rationale | | 2 | Physical activity (PA) in children and adolescents with visual impairments (VI) is imperative for physical and mental health. However, there has been reduced PA among them owing to functional impairment, resulting in declining health and quality of life. Prior studies have highlighted parental support as a key social determinant of PA in typically developing children and adolescents. However, its specific mechanisms, particularly the mediating role of exercise self-efficacy in children and adolescents with VI remain insufficiently studied. Therefore, we aimed to investigate the associations between parental support and moderate-to-vigorous physical activity (MVPA) levels in children and adolescents with VI, as well as the mediating role of exercise self-efficacy. **(page3-6)** |
| Objectives | | 3 | To examine the associations between parental support and moderate-to-vigorous physical activity (MVPA) levels in children and adolescents with visual impairment (VI), and to investigate whether exercise self-efficacy mediates these relationships. **(page6)** |
| Methods (page6-10) | | | |
| Study design | | 4 | Cross-sectional study. |
| Setting | | 5 | Six special education schools in East China (Jiangsu province, Anhui province, Shanghai province). Data collection was conducted at participating schools during school hours from September to December 2024 by trained research assistants and teachers. **(page6)**  Describe the setting, locations, and relevant dates, including periods of recruitment, exposure, follow-up, and data collection |
| Participants | | 6 | A total of 340 children and adolescents with VI aged 7-18 (mean age 13.80±2.94 years) from six specialized schools were recruited through convenience sampling in East China. The diagnosis of visual disability was in accordance with the Chinese national standard "Classification and Grading of Disability for Persons with Disabilities". Inclusion criteria: ① Age 7–18 years; ② No cognitive impairments; ③ No other significant physical defects, able to complete the tests with assistance from parents and teachers; ④ Voluntarily participated in this study and provided signed parental informed consent forms. A total of 34 participants were excluded, and finally, 306cases were included in the analysis. All participants were enrolled voluntarily, and written informed consent was obtained from their parents or guardians. **(page6)**  *Cross-sectional study*—Give the eligibility criteria, and the sources and methods of selection of participants |
|  |  |  |  |
| Variables | | 7 | The primary outcome variable was %MVPA. The main exposure variable was parental support for PA. The mediating variable was exercise self-efficacy. **(page7-9)**  Clearly define all outcomes, exposures, predictors, potential confounders, and effect modifiers. Give diagnostic criteria, if applicable |
| Data sources/ measurement | | 8* | Data on physical activity were obtained through accelerometer measurements. MVPA was measured objectively using GT3X+ accelerometer worn for 7 consecutive days. It was defined as ≥2296 cpm, based on the cut-points established by Evenson et al. (2008), and the percentage of MVPA time (%MVPA) relative to total wear time was derived.  Data on parental support were collected using the Parental Support Questionnaire. Parental support was measured using five items from the Parental Support Questionnaire developed by Liu et al. (2017). Each item was rated on a Likert scale, and a total score was calculated. A higher total score indicates greater parental support.  Data on exercise self-efficacy were obtained from the Exercise Self-Efficacy Scale. Exercise Self-Efficacy was measured using six items from the Exercise Self-Efficacy(ESE) Scale developed by Pirasteh (2008a). Each item was rated on a Likert scale, and a total score was calculated. A higher total score indicates greater exercise self-efficacy.  Data on age, sex, and grade level were derived from a basic information survey completed by the participants. Data on height , weight and degree of visual disability were obtained from school records. BMI was calculated based on height and weight,  and the degree of visual disability was classified according to the Chinese national standard "Classification and Grading of Disability for Persons with Disabilities".  **(page7-9)**  For each variable of interest, give sources of data and details of methods of assessment (measurement). Describe comparability of assessment methods if there is more than one group |
| Bias | | 9 | **Control of measurement bias:** Objective measurements were conducted using the ActiGraph GT3X+ accelerometer to avoid recall bias and social approval bias that may exist in self-reported physical activity. All questionnaires adopted mature scales that had been tested for reliability and validity in similar populations. **Control of selection bias:** Clear inclusion criteria (aged 7 to 18, no cognitive impairment, no other obvious physiological defects, and voluntary participation) was established to ensure the representativeness of the sample and the comparability of the research results. **(page7-8)**  Describe any efforts to address potential sources of bias |
| Study size | | 10 | The sample size for this study was calculated based on classical rules for determining sample size in structural equation modeling. Taking a common mediation model as an example, the free parameters in this study include: 9 factor loadings (4 estimated for the first latent variable with 5 items, and 5 estimated for the second latent variable with 6 items), 12 measurement error variances, 2 latent variable variances, 1 covariance between latent variables, and 3 structural path coefficients, totaling 27 free parameters. According to the recommended 10-times rule, the required sample size is at least 27 × 10 = 270 cases. Considering potential issues such as equipment malfunction or invalid data in physical activity measurement, missing data in questionnaires, statistical power, model stability, and the accessibility of the study population, this study planned to measure 340 participants, yielding a final effective sample size of 304 cases to ensure the robustness of model estimation and the statistical power for testing mediation effects. **(page9-10)** |
| Quantitative variables | | 11 | Parental support, physical activity, and exercise self-efficacy were all included in the structural equation model analysis as continuous variables. Age groups: divided into 7-12 years old (primary school group) and 13-18 years old (middle school group). This grouping was based on the phased characteristics of the physical and mental development of children and adolescents. **(page8-10)**  Explain how quantitative variables were handled in the analyses. If applicable, describe which groupings were chosen and why |
| Statistical methods | | 12 | **Descriptive analysis：**Data processing and analysis were performed using SPSS 24.0 software. The Shapiro-Wilk test was applied to assess the normality of all continuous variables. Variables that conformed to a normal distribution were reported as mean ± standard deviation (x̄ ± s). **Comparison of differences：**Gender and age differences in all outcomes were examined using independent t-tests. **correlation analysis：**The correlations between PA, exercise self-efficacy and parental support were examined using the Pearson’s partial correlation coefficients, controlling for gender and age, with statistical significance defined as α< 0.05. **Analysis of Mediating Effects：**Structural equation modeling (SEM) was performed with AMOS 26.0 to evaluate the hypothesized model and to examine the potential mediating role of exercise self-efficacy in the relationship between parental support and MVPA. Confirmatory factor analysis was performed on the latent variables (parental support, exercise self-efficacy) to evaluate the goodness of fit of the measurement model. Based on the good fit of the measurement model, a structural equation model is constructed to test the mediation path. The robust maximum likelihood estimation method is adopted for parameter estimation. The bias-corrected Bootstrap method was used to repeat the sampling 5000 times, and the 95% confidence interval for the mediating effect was calculated. If the confidence interval does not include 0, the mediating effect is statistically significant. **(page9-10)**  (*a*) Describe all statistical methods, including those used to control for confounding |
|  |  |  | We did not conduct formal subgroup analyses or test for interactions, as the study was powered primarily to detect the overall mediation effect rather than moderation effects.  (*b*) Describe any methods used to examine subgroups and interactions |
|  |  |  | (1) Accelerometer data: The criteria for valid data were a minimum wear time of 10 hours per day, including at least 3 valid weekdays and 1 valid weekend day. Individuals who did not meet these criteria were excluded from the analysis.  (2) Questionnaire data: If less than 20% of the items in a single questionnaire were missing, the missing values were imputed using the mean of the items that were answered. If missing items reached or exceeded 20%, the case was excluded. **(page9-10)**  (*c*) Explain how missing data were addressed |
|  |  |  | We did not apply sampling weights or adjust for clustering, as participants were recruited through convenience sampling and the primary aim was to examine mechanistic relationships rather than produce population prevalence estimates.  (*d*) *Cohort study*—If applicable, explain how loss to follow-up was addressed  *Case-control study*—If applicable, explain how matching of cases and controls was addressed  *Cross-sectional study*—If applicable, describe analytical methods taking account of sampling strategy |
|  |  |  | We did not conduct formal sensitivity analyses due to limited sample size and model parsimony. Key assumptions (e.g., linearity, normality of residuals) were checked and met.  (*e*) Describe any sensitivity analyses |
| Results (page6，10-15) | | | |
| Participants | 13* | A total of 340 participants were recruited and included in the study, completing the surveys and tests. After data exclusions, a final total of 306 participants were included in the structural equation model analysis. **(table1, page6)**  (a) Report numbers of individuals at each stage of study—eg numbers potentially eligible, examined for eligibility, confirmed eligible, included in the study, completing follow-up, and analysed | |
|  |  | Six participants were excluded due to instrument failure resulting in missing physical activity data. Another 18 participants were excluded due to invalid accelerometer data (defined as wearing the device for fewer than 2 weekdays and 1 weekend day, or less than 8 hours per day). Additionally, 10 participants were excluded due to invalid questionnaire data. Ultimately, 306 valid datasets were included in the analysis, comprising 163 boys and 143 girls.  (b) Give reasons for non-participation at each stage | |
|  |  | (c) Consider use of a flow diagram | |
| Descriptive data | 14* | Children and adolescents with VI aged between 7 and 18 years were initially recruited from six special education schools in the East China. The participants, who included individuals with total blindness and low vision, were from three schools in Jiangsu Province, two in Anhui Province, and one in Shanghai City. (**table1, page6**)  (a) Give characteristics of study participants (eg demographic, clinical, social) and information on exposures and potential confounders | |
|  |  | (b) Indicate number of participants with missing data for each variable of interest | |
|  |  | (c) *Cohort study*—Summarise follow-up time (eg, average and total amount) | |
| Outcome data | 15* | *Cohort study*—Report numbers of outcome events or summary measures over time | |
|  |  | *Case-control study—*Report numbers in each exposure category, or summary measures of exposure | |
|  |  | This sample of participants with VI accumulated 80.38% of wear time in sedentary behavior and only 5.11% (43.62 min/day) in MVPA. Parental support and its sub-dimensions were positively correlated with %MVPA(r=0.173-0.306, p<0.05). The direct effects of parental support on physical activity and exercise self-efficacy were 0.29 and 0.24, respectively, and self-efficacy had a direct effect of 0.35 on %MVPA. The indirect effect value of parental support on the effect of %MVPA was 0.085, 95% CI [0.039, 0.146] excluding 0 (p<0.01), indicating a significant mediated effect. (**page10-12**)  *Cross-sectional study—*Report numbers of outcome events or summary measures | |
| Main results | 16 | Table 2 reports relationship between parental support, exercise self-efficacy and %MVPA (95% CI) after adjusting for age, gender and daily valid wear time.  (*a*) Give unadjusted estimates and, if applicable, confounder-adjusted estimates and their precision (eg, 95% confidence interval). Make clear which confounders were adjusted for and why they were included. (**page10-12**) | |
|  |  | (*b*) Report category boundaries when continuous variables were categorized | |
|  |  | (*c*) If relevant, consider translating estimates of relative risk into absolute risk for a meaningful time period | |
| Other analyses | 17 | Report other analyses done—eg analyses of subgroups and interactions, and sensitivity analyses | |
| Discussion (page12-16) | | | |
| Key results | 18 | Significant positive correlations were observed between parental support and MVPA (r=0.306, P<0.01), as well as between exercise self-efficacy and MVPA (r=0.385, P<0.01). Parental support exerted a direct effect on MVPA levels in children and adolescents with VI (β=0.293, P<0.001), while exercise self-efficacy also demonstrated a direct effect (β=0.348, P<0.001).The total effect of parental support on MVPA was 0.379 (95%CI [0.226, 0.523], P<0.01). Notably, parental support indirectly influenced MVPA through its mediating effect on exercise self-efficacy, with a mediated effect value of 0.085, 95% CI [0.039, 0.146], P<0.01), indicating a statistically significant mediating role of exercise self-efficacy. The partial mediation structural model accounted for 26.3% of the variance in MVPA. **(page12-15)**  Summarise key results with reference to study objectives | |
| Limitations | 19 | This study has several limitations. Firstly, the sample was drawn from six cities, which may not fully represent the diversity of children and adolescents with visual impairments; future studies should expand recruitment to include multiple regions and school types. Thirdly, the cross-sectional design precludes causal inferences regarding parental support and physical activity (PA); longitudinal or experimental approaches (e.g., assessing PA changes following parental support interventions) are needed to establish temporal relationships. Finally, by focusing primarily on parental support and self-efficacy, other potential confounders (e.g., school policies, peer influence) may have been overlooked. Future research should adopt a socioecological framework to examine multilevel determinants or employ SEM to analyze complex pathways. **(page16)**  Discuss limitations of the study, taking into account sources of potential bias or imprecision. Discuss both direction and magnitude of any potential bias | |
| Interpretation | 20 | Give a cautious overall interpretation of results considering objectives, limitations, multiplicity of analyses, results from similar studies, and other relevant evidence | |
| Generalisability | 21 | Discuss the generalisability (external validity) of the study results | |
| Other information | | | |
| Funding | 22 | This research was funded by Social Science Foundation of Jiangsu Province (NO.22TYB004, and Key grant Project of Humanities and Social Science Foundation of Department of Education of Jiangsu of China (NO. 2022SJZD085). **(page16)**  Give the source of funding and the role of the funders for the present study and, if applicable, for the original study on which the present article is based | |

*Give information separately for cases and controls in case-control studies and, if applicable, for exposed and unexposed groups in cohort and cross-sectional studies.

**Note:** An Explanation and Elaboration article discusses each checklist item and gives methodological background and published examples of transparent reporting. The STROBE checklist is best used in conjunction with this article (freely available on the Web sites of PLoS Medicine at http://www.plosmedicine.org/, Annals of Internal Medicine at http://www.annals.org/, and Epidemiology at http://www.epidem.com/). Information on the STROBE Initiative is available at www.strobe-statement.org.
